# Metal-organic frameworks-loaded indocyanine green for enhanced phototherapy: a comprehensive review

**DOI:** 10.3389/fbioe.2025.1601476

**Published:** 2025-05-27

**Authors:** Yutao Zou, Qiuyun Zhang, Jiayi Chen, Weiqi Wang, Xiaohua Zheng

**Affiliations:** ^1^ The People’s Hospital of Danyang, Affiliated Danyang Hospital of Nantong University, Danyang, Jiangsu, China; ^2^ School of Pharmacy, Nantong University, Nantong, Jiangsu, China

**Keywords:** indocyanine green, metal-organic frameworks, photodynamic therapy, photothermal therapy, cancer therapy

## Abstract

Indocyanine green (ICG) is a small molecule approved by the U.S. Food and Drug Administration (FDA) for liver function imaging and angiography. ICG can be used not only for near-infrared imaging but also for photodynamic and photothermal therapy. However, the hydrophilicity of ICG leads to a relatively short blood circulation time, and it is easily cleared by organs such as the liver. Moreover, it lacks the targeting ability to the diseased sites. By using the natural porous metal-organic frameworks (MOFs) as the carrier, high-efficiency loading of ICG molecule can be achieved, which has significantly broadened its biomedical applications. This review comprehensively summarizes the research work in recent years regarding the utilization of MOF as a carrier to load ICG in the bioapplication such as malignant cancer inhibition, antibacterial treatment, and the treatment of Alzheimer’s disease. It focuses on summarizing the design concepts of different types of MOF carriers for loading ICG molecules. Meanwhile, it emphasizes the enhanced therapeutic effects achieved when multiple treatment modalities realized through post-modification are combined with ICG-mediated phototherapy. It is expected that through the summary of this review, the biomedical applications of ICG in the field of disease treatment can be further promoted.

## 1 Introduction

The diversity and heterogeneity of malignant tumors necessitate substantial human and financial investments in research aimed at improving therapeutic strategies, thereby enhancing the quality of life (Marusyk et al., 2012; Dagogo-Jack and Shaw, 2018; Black and McGranahan, 2021). Among various treatment modalities, phototherapy has attracted considerable attention due to its low cost and minimal invasiveness (Correia et al., 2021; Niculescu and Grumezescu, 2021; Ouyang et al., 2022; Jiang et al., 2023). Phototherapy can be specifically categorized into photodynamic therapy (PDT) and photothermal therapy (PTT) (Lopes et al., 2022; Nguyen et al., 2022; Luo and Gao, 2023). Both approaches rely on photoactive materials for energy transfer, conversion, or electron transfer, generating heat or reactive oxygen species (ROS) (Zheng et al., 2021; Ouyang et al., 2024; Singh et al., 2024). PDT induces apoptosis by producing toxic ROS that inhibit cancer cell proliferation, while PTT achieves cancer cell necrosis or apoptosis by elevating local temperatures above physiological levels, leading to the inactivation of biomacromolecules within cancer cells (Ma and Xue, 2021; Pham et al., 2021; Alamdari et al., 2022; Yang et al., 2022; Jiang et al., 2023; Kim et al., 2023).

Phototherapy requires both an excitation light source and photoactive materials (Amos-Tautua et al., 2019; Tian J. et al., 2020; Alle et al., 2022; Xu et al., 2023). A wide variety of photoactive materials have been explored, with organic molecules such as porphyrins (Yu et al., 2020), BODIPY (Turksoy et al., 2019), IR780 (Bai et al., 2024; Gao et al., 2024) and cyanine dyes (Lange et al., 2021; Zhang J. et al., 2024) being extensively studied. Unlike porphyrins and BODIPY with simple structures, which have absorption wavelength ranges in the visible light region, cyanine molecules, due to their more extensive conjugated structures, can achieve an absorption wavelength range of 650–1,350 nm (Yuan et al., 2025). During the phototherapy process, a light source is required to excite the photoactive materials. Shorter wavelengths not only fail to penetrate body tissues but are also absorbed by the body. Larger wavelengths, especially those in the first and second near-infrared regions, generally alleviate the limitations of these issues on the biomedical applications of photoactive materials that respond to light (Chen et al., 2023b; Luo and Gao, 2023; Li et al., 2024; Schmidt et al., 2024). Therefore, cyanine-based photoactive materials possess significant advantages and have been applied and developed in a variety of phototherapy systems for the diagnosis and treatment of multiple diseases (Lange et al., 2021). The structure of classical cyanine dyes is generally formed by two nitrogen-containing heterocycles connected by a conjugated polymethine chain. Due to the fact that the substituents on the nitrogen in the polymethine chain and the heterocyclic ring can be designed into different structures according to different raw materials, there is a wide variety of cyanine- based materials with different absorption and emission wavelengths. A common classification method is to divide them into Cy2, Cy3, Cy3.5, Cy5, Cy5.5, Cy7, and Cy7.5. Indocyanine green, a member of the Cy7 series, is particularly noteworthy as the only FDA-approved cyanine dye for clinical use (Kumari et al., 2024). With maximal absorption around 780 nm within the first near-infrared window (NIR-I, 700–900 nm), ICG could minimize tissue absorption and maximize energy delivery to deeper-seated targets (Wang H. et al., 2018). Additionally, ICG emits fluorescence suitable for imaging purposes and exhibits both PDT and PTT effects under laser irradiation, simplifying therapeutic protocols (Boni et al., 2015; Li et al., 2016; Hu et al., 2018; Wang Q. et al., 2020; Zhao et al., 2022). However, ICG’s hydrophilicity leads to rapid clearance from the bloodstream and challenges in achieving targeted delivery (Beziere et al., 2015; Porcu et al., 2016; Egloff-Juras et al., 2019; Gowsalya et al., 2021). Therefore, developing carriers to load ICG is essential for optimizing its therapeutic efficacy.

Carriers used for the delivery of ICG molecules in phototherapy are diverse, including silica nanoparticles (NPs) (Gowsalya et al., 2022; Kang et al., 2022; Li J. et al., 2022; Sun et al., 2022; Ma et al., 2024; Qi et al., 2024), organic polymers (De Clerck et al., 2022; Du et al., 2022; Wang K. et al., 2022; Wu et al., 2023; Zhang et al., 2023), dendrimers (Guo et al., 2022; Ouyang et al., 2023), proteins (Xu et al., 2022b; Jang et al., 2023; Xiong et al., 2024; Zhou et al., 2024; Roy et al., 2025), liposomes (Wang R. et al., 2022; Shan et al., 2023; Kim et al., 2024; Liao et al., 2024), and cell membranes (Chi et al., 2022; Pan et al., 2022; Li et al., 2023; Liu P. et al., 2024). Comparatively speaking, MOFs offer unique advantages as carrier materials (Fatima et al., 2023; Lin et al., 2023; Hayat et al., 2024; Vikal et al., 2024; Zhang Q. et al., 2024; Zou et al., 2024a; Zou et al., 2024b; Gong et al., 2025). Firstly, MOFs are compounds with porous structures formed by the coordination of metal ions or clusters with polycarboxylate ligands or imidazole-like molecules (Sun et al., 2020; Wang Y. et al., 2020; Lawson et al., 2021; Ding et al., 2022; Maranescu and Visa, 2022; Rabiee, 2023; Sun et al., 2023). The porous structure of MOFs enables highly efficient loading of ICG, with encapsulation efficiencies exceeding 70%, which is significantly higher than those of liposomes and polymeric nanoparticles (Wang K.-F. et al., 2023). Secondly, MOFs can protect ICG from photodegradation and rapid clearance, thereby prolonging circulation time in the bloodstream. Thirdly, MOFs themselves are extensively studied as novel biomaterials due to their excellent biocompatibility and easily modifiable surfaces, which can endow drug systems with targeting capabilities that are crucial for nanomedicine efficacy (Yang and Yang, 2020; Ge X. et al., 2022; Haider et al., 2022; Rabiee et al., 2022; Wiśniewska et al., 2023). Therefore, the development of MOFs for loading ICG molecules in phototherapy research holds great application potential (Golmohamadpour et al., 2018; Wang T. et al., 2018; Yang et al., 2018; Cheng Y. et al., 2021; Fan et al., 2021). This approach not only maximizes the therapeutic benefits of ICG but also enhances its stability and targeting efficiency, making it a promising direction in the field of cancer therapy (Xu et al., 2021; Hao et al., 2023; Lin et al., 2024; Lu et al., 2024).

**FIGURE 1:**
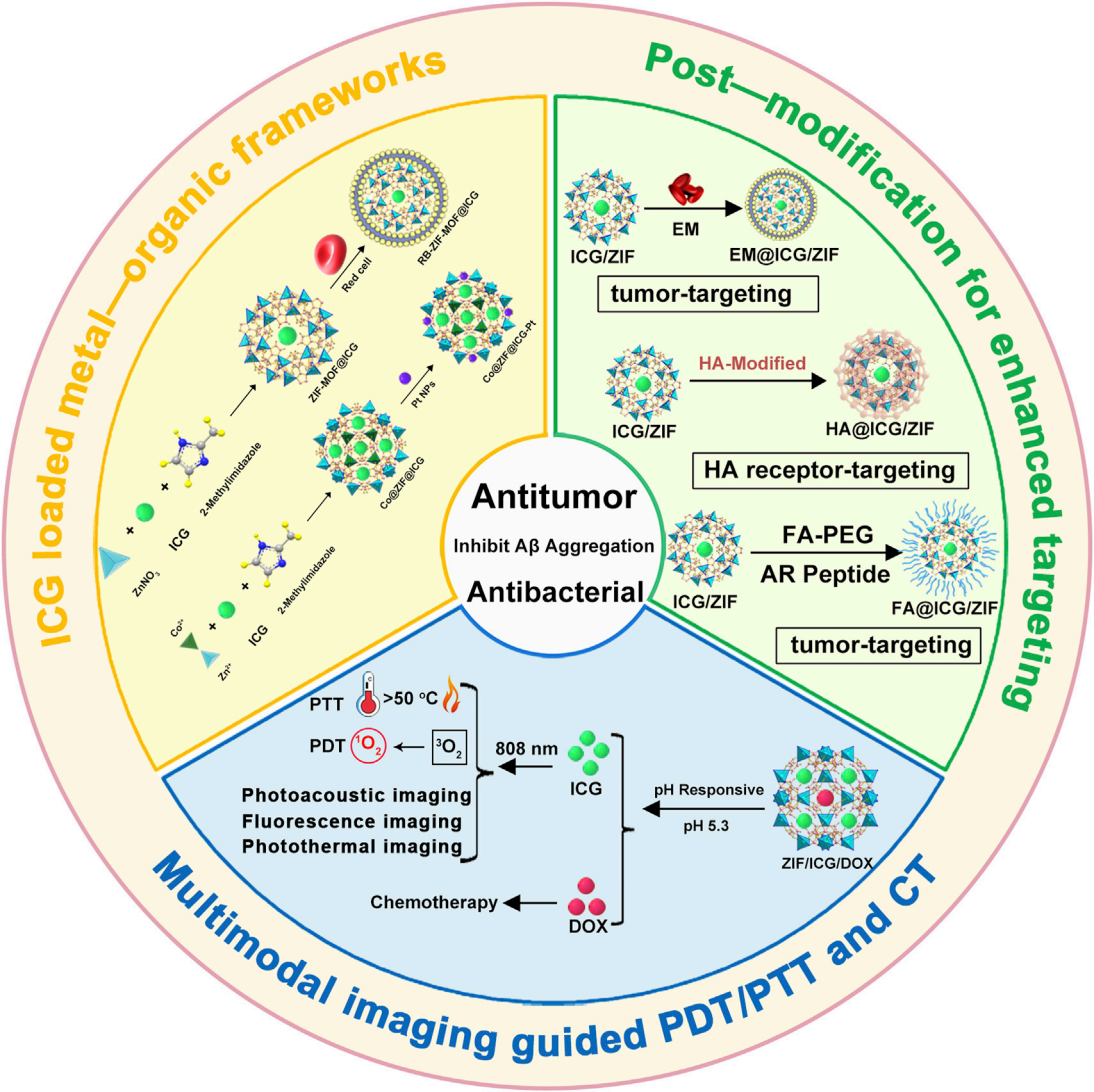
Schematic illustration of the preparation of ICG-loaded MOFs materials (ZIF-MOF@ICG, RB-ZIF-MOF@ICG, Co@ZIF@ICG and Co@ZIF@ICG-Pt) and various surface post-modification with red blood cell membrane, folic acid, or hyaluronic acid for enhanced targeting towards tumor sites for combined photodynamic and photothermal therapy. Schematic illustration of the multifunctional ZIF/ICG/DOX material releasing ICG and DOX molecules in response to the pH of the tumor microenvironment, enabling multimodal imaging-guided combination therapy of phototherapy and chemotherapy. EM, erythrocyte membrane; HA, hyaluronic acid; FA, folic acid; AR, AREYGTRFSLIGGYR; DOX, doxorubicin.

This review summarizes recent advancements in utilizing various MOFs (e.g., Zeolitic Imidazolate Frameworks (ZIF), UiO (University of Oslo), (Porous Coordination Networks) PCN, (Materials of Institute Lavoisier) MIL series) as carriers for loading ICG, focusing on multimodal imaging-guided combination therapies (Figure 1). It elaborates on the design principles of MOFs@ICG systems and discusses the synergistic effects of ICG-mediated phototherapy combined with other therapeutic modalities, including chemotherapy, chemodynamic therapy (CDT), and immunotherapy. Furthermore, it highlights strategies for post-modifications of MOFs to improve blood circulation and tumor-targeting efficiency, aiming to provide valuable insights for the clinical application of ICG-based phototherapeutics.

## 2 Design rationale and advantages for MOFs as carriers for ICG

**FIGURE 2:**
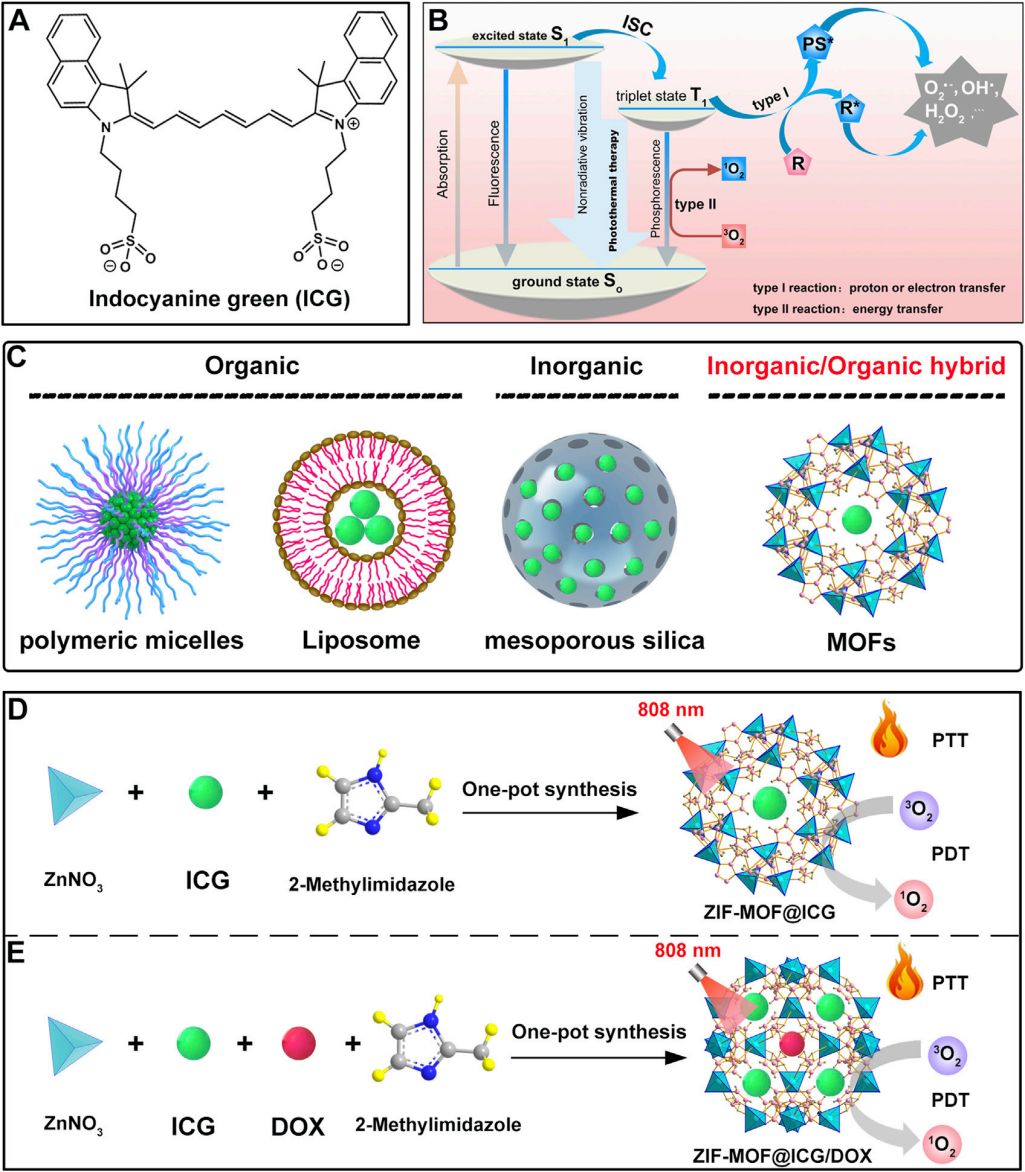
**(A)** Molecular structure of ICG. **(B)** Schematic illustration of the mechanism of ICG for PDT and PTT. **(C)** Schematic illustration of various carriers, including polymeric micelles, liposomes, mesoporous silica, and MOFs, as carriers for ICG. **(D)** Schematic illustration of the preparation of ICG-loaded ZIF-MOFs and their PDT and PTT effects under 808 nm light irradiation. **(E)** Schematic illustration of the preparation of ZIF-MOFs loaded with ICG and doxorubicin, and their PDT and PTT effects induced by 808 nm light irradiation as well as combined chemotherapy effects.

ICG molecules possess an extensive conjugated structure, resulting in a significant absorption wavelength (Figure 2A) (Yuan et al., 2025). Additionally, their unique structural composition endows them with excellent photochemical properties (Figure 2B) (Kumari et al., 2024). At room temperature, ICG exhibits a high molar extinction coefficient of 224,000 M^-1^cm^-1^ in DMSO, demonstrating its strong light absorption capability and indicating that it is a highly efficient photoactive material (Li and Smith, 2021). Therefore, ICG molecules can be easily excited by photons, absorb energy, and transition to the excited state (Kumari et al., 2024). Excited-state ICG molecules can undergo intersystem crossing to reach a triplet state, during which energy can be transferred to oxygen molecules to generate singlet oxygen. Furthermore, both singlet and triplet excited states of ICG can dissipate energy as heat through non-radiative vibration, contributing to photothermal effects (Figure 2B).

As a biocompatible material with excellent photochemical properties, ICG has been widely developed in combination with various carrier systems. These carrier materials can generally be divided into three categories: organic carriers (polymeric micelles and liposomes) (Du et al., 2022; Wang R. et al., 2022), inorganic carriers (mesoporous silica) (Qi et al., 2024), and hybrid inorganic/organic carriers such as MOFs (Figure 2C). Organic carriers like polymeric micelles or liposomes are extensively studied due to their good biocompatibility and biodegradability. However, when these carriers are combined with ICG, there is a risk of premature leakage (Jian et al., 2015). As an inorganic carrier, mesoporous silica degrades slowly, which may lead to chronic inflammation and long-term toxicity in the body (Croissant et al., 2018). In contrast, as hybrid inorganic/organic materials, MOFs offer unique advantages by combining the functionalities of organic ligands and inorganic metals. Most MOFs can degrade under the action of phosphate, ensuring they do not accumulate in the body for extended periods. More notably, ZIF-MOFs, a subclass of MOFs, can slowly degrade in the weakly acidic environment of cancer cells (Jiang F. et al., 2022). It is well-known that the heterogeneity of tumor tissues makes it difficult for a single treatment method to achieve optimal therapeutic effects (Fan et al., 2017). Combining multiple treatment modalities allows for complementary advantages, making combination therapy a promising approach (Fan et al., 2017). The pH-responsive release mechanism of ZIF-MOFs provides a guarantee for the effective performance of various treatment strategies. Furthermore, carriers like polymers or silica spheres typically lack significant biological functions. In contrast, MOFs not only serve as carriers but also provide additional therapeutic benefits. For instance, the metal components in MOFs can contribute to imaging or CDT, while the organic ligands involved in their structure can enable phototherapy (Jiang F. et al., 2022). Additionally, the structural diversity and ease of modification of MOFs offer possibilities for designing customized nanomedicine systems. Therefore, MOFs hold substantial research value as carrier materials for ICG.

In this review, various types of MOFs used for ICG loading are discussed, including ZIF, UiO, MIL, and PCN series (Liu H. et al., 2022; Wang K.-F. et al., 2023; Jiang et al., 2024; Liu Y. et al., 2024). When designing combination therapies that simultaneously load ICG and chemotherapeutic agents such as doxorubicin (DOX), the ZIF series is often chosen due to its relatively mild synthesis conditions and ability to degrade under the acidic microenvironment of cancer cells, facilitating drug release (Liu B. et al., 2022; Chen et al., 2023a). Here, we provide a brief overview of using ZIF-8 as a carrier for ICG alone (Figure 2D) or in combination with DOX (Figure 2E). The preparation of ZIF-MOFs loaded with ICG can be efficiently achieved via a one-pot method. Specifically, zinc nitrate, 2-methylimidazole, and ICG solutions in methanol are prepared separately. The ICG solution is then added to the zinc nitrate solution, followed by the dropwise addition of the 2-methylimidazole solution. The mixture is stirred for 1 h, after which the product is collected by centrifugation and washing, yielding ZIF-MOF@ICG material (Figure 2D). For co-loading with DOX, an additional DOX solution is included in the mixture, followed by similar post-processing steps to obtain ZIF-MOF@ICG/DOX material (Figure 2E). This approach for loading ICG or DOX into MOFs is straightforward and effective, requiring no complex organic synthesis or post-treatment procedures, thus holding great potential for practical applications.

A variety of MOF materials have been utilized to load ICG and other drug molecules, achieving effective treatment for multiple diseases (Liu B. et al., 2022). MOFs serve not only to extend the circulation stability of drugs like ICG in the bloodstream but also enhance their accumulation in cancer cells through the enhanced permeability and retention effect (Jiang F. et al., 2022). Moreover, MOFs can be further modified with various active targeting molecules, such as small molecules, polymers, or cell membranes, to improve their targeting efficiency towards cancer cells (Ge T. et al., 2022; Jiang Z. et al., 2022; Wang K.-F. et al., 2023). To systematically summarize and compare relevant research efforts, this review provides a detailed list in Table 1, which includes the composition of materials, imaging modalities, and the principles of combination therapy from representative studies in recent years. This table compares the types of materials, their structures, the types of cancer cells treated, or other diseases across multiple different systems. It highlights the advantages of MOFs as carriers for ICG, provides new candidates for the treatment of various diseases, and offers new references for the further clinical application of ICG.

**TABLE 1:** Synthesis of nMOFs as carriers for ICG for bioapplication.

Material/targeting ligands	Structure		Bioapplication mechanism/Therapeutic advantages	References
	Nanoscale MOF	Physicochemical properties		
ICG@RB-MOF, erythrocyte membrane	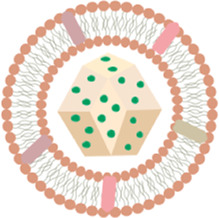	122 ± 1.3 nm, −4.14 mV, rhombic dodecahedron	ICG-PTT, immune escape, prolonged blood circulation, pancreatic cancer cells	Wang et al. (2023a)
ICG@ZIF8(Al)	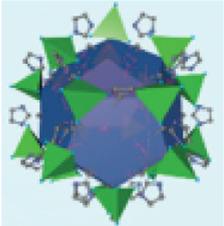	∼100 nm, dodecahedron	ICG-PTT, Al NPs-immunotherapy, 4T1 cancer cells	Li et al. (2022a)
CZIP Co/ZIF-8/ICG-Pt	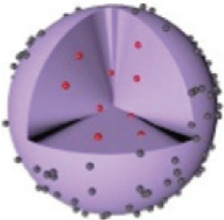	100–150 nm, −15.57 mV, mono-dispersed NPs	ICG-PDT, Co^2+^-CDT, Pt NPs for O_2_ generation, HeLa cells	Jiang et al. (2022a)
ICG@Cu_2-X_Se-ZIF-8	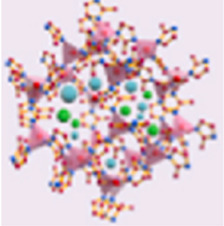	250–300 nm, ∼1.5 mV, mono-dispersed NPs	ICG-PTT, Cu^+^-CDT, Se-regulates Selenoprotein, MDA-MB-231 cells	Zou et al. (2022)
UCNPs@ZrMOF@ICG	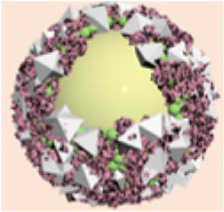	∼45 nm, +5 mV mono-dispersed NPs	PDT (1,532 nm), PTT (808 nm), HCT116 cancer	Jiang et al. (2024)
DOX/Z-ICG-FA, folic acid-tumor targeting	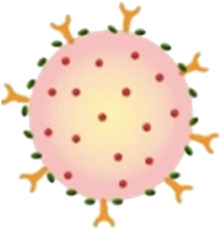	188 nm, −8.6 mV, dodecahedron	ICG-PTT, DOX-chemotherapy, MCF-7 cancer cell	Liu et al. (2022a)
5-Fu/ICG@ZPZ, zoledronic acid-bone-targeting	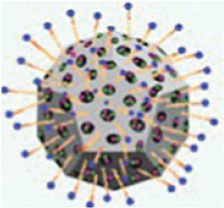	113 nm (DLS), 0.198 (PDI), mono-dispersed NPs	ICG-PTT, 5-Fu-chemotherapy, MCF-7 cancer cells	Ge et al. (2022a)
ICG&DHA@ZIF-8	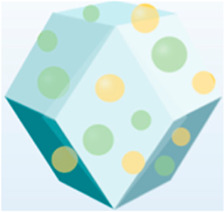	137.5 ± 20.5 nm (DLS), *−*19.4 mV, polyhedron shape	ICG-PDT, DHA-chemotherapy, HepG2 cancer cells	Chen et al. (2022)
ICG/Cyt c@ZZF-8, zoledronic acid-bone-targeting	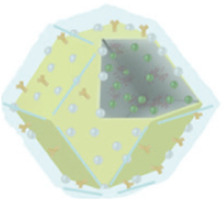	213.29 nm, 0.075 (PDI), −14.9 mV	ICG-PDT, Cyt c-an anticancer protein, Cyt c (H_2_O_2_ to O_2_), 4T1 cancer cell	Jiang et al. (2022b)
OIMH NPs, hyaluronic acid	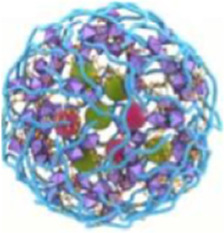	127 nm, −30 mV, spherical morphology	ICG-PTT, Oxaliplatin-chemotherapy Immunogenic cell death, CT26 cancer cells	Liu et al. (2022b)
AR-ZS/ID-P, AR peptide (tumor-targeting)	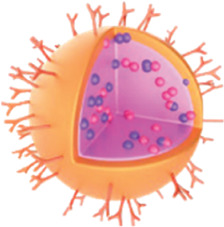	135 nm, −8.88 mV, mono-dispersed NPs	ICG-PDT, PTT, DOX-chemotherapy, MCF-7 cancer cells	Chen et al. (2023a)
MIL-88-ICG@ ZIF-8-DOX	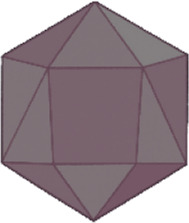	MIL-88 (octahedron shape, 100 nm)	ICG-PDT, PTT, DOX-chemotherapy, 4T1 cancer cells	Wu et al. (2020a)
CIDF, folic acid-tumor targeting	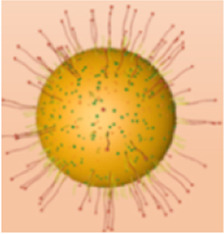	155 nm, ∼9 mV, spherical structure	ICG-PDT, PTT, DOX-chemotherapy, Fe^3+^ (GSH to GSSG), Fe^3+^ (H_2_O_2_ to O_2_), HeLa cells	Liu et al. (2024b)
ZIF-8/DA-0.5/ICG	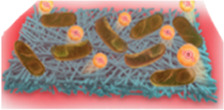	∼295 nm, polyhedral morphology	ICG-PDT, PTT, antibacterial phototherapeutics	Gao et al. (2022)
PCN−222@ICG@RVG, brain-targeting peptide RVG	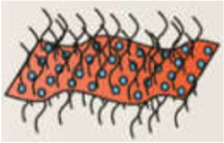	∼120 nm, nanosheet	ICG-PDT, PTT, inhibition of Aβ aggregation	Wang et al. (2022b)

Abbreviations: GSH (glutathione); RB (red blood); DHA (dihydroartemisinin); 5-Fu (5-fluorouracil).

## 3 Erythrocyte membrane-coated ICG@MOF for phototherapy

**FIGURE 3:**
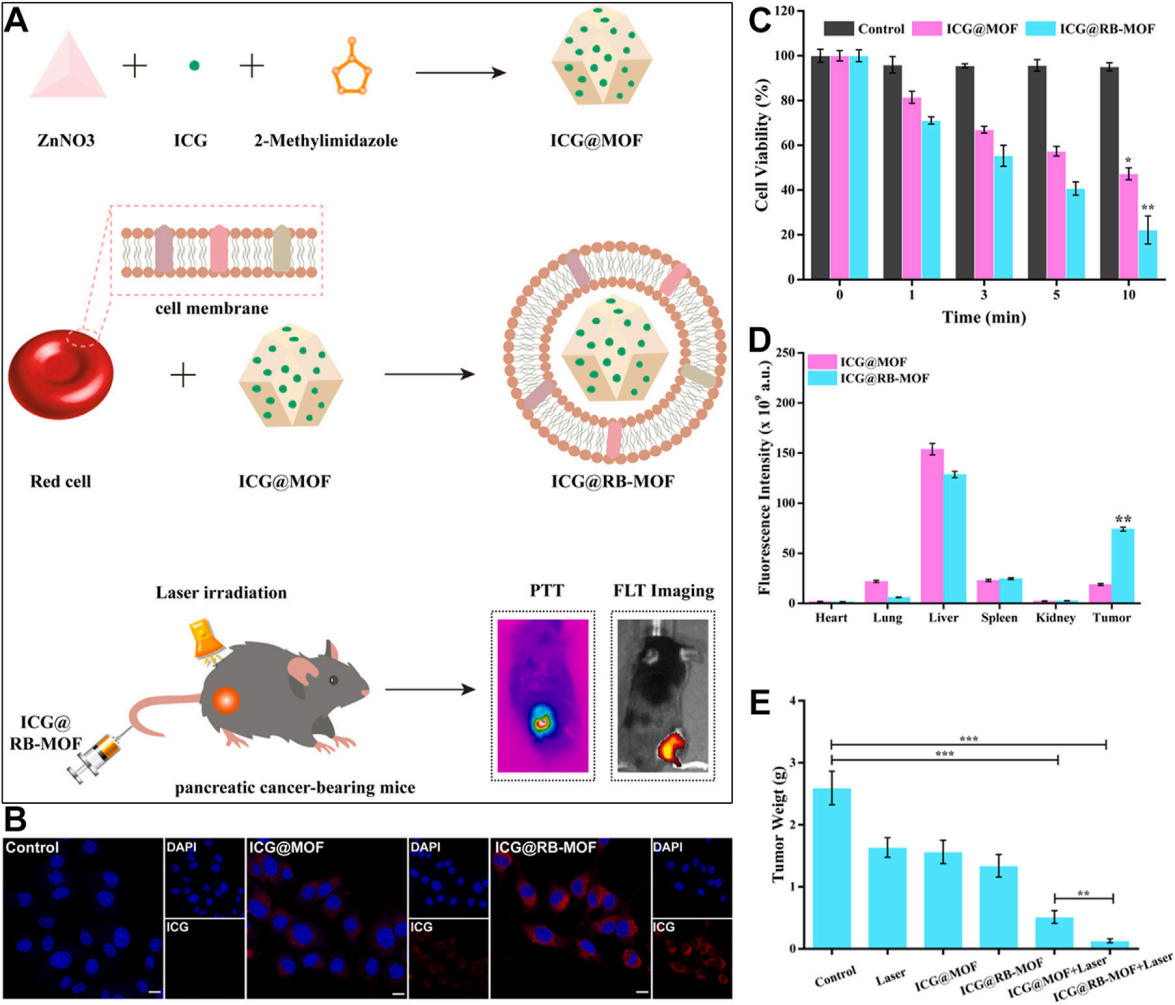
**(A)** Preparation process of ICG@MOF, ICG@RB-MOF and its theranostic application. **(B)** Comparison of endocytosis efficiency of ICG@MOF and ICG@RB-MOF detected by CLSM (scale bar: 10 µm). **(C)** Cell viability after incubation with ICG@MOF and ICG@RB-MOF under laser irradiation. **(D)** Biodistribution of ICG@MOF and ICG@RB-MOF. **(E)** Tumor weight changes after different treatments. Reproduced with permission from (Wang K.-F. et al., 2023). Copyright (2022), Elsevier.

As a malignant tumor associated with the digestive system, pancreatic cancer severely influences human health due to its high aggressiveness and low survival rate (Mizrahi et al., 2020; Hu J. X. et al., 2021). The inherent invasive characteristics of pancreatic cancer limit the effectiveness of clinical surgical treatments. Moreover, conventional chemotherapy drugs face limitations in treatment efficacy because of drug resistance (Hani et al., 2021; Bukhari, 2022; Tarannum and Vivero-Escoto, 2022). With advancements in technology, researchers have discovered an increasing number of treatment methods. Among these, PTT stands out as a non-invasive approach (Liu et al., 2019; Tekade et al., 2023; Xie et al., 2024). The PTT modality uses photothermal conversion agents to transform light energy into heat, raising the temperature in the affected area, which leads to the deactivation of various metabolism-related proteins, causing cell death (Liu et al., 2019). There are many types of photothermal conversion agents, among which ICG, a biocompatible photoactive material, has shown great potential. However, its short circulation time in the bloodstream and poor accumulation at tumor sites hinder its full therapeutic effect. To address this issue, Wang et al. developed ICG@MOF by mixing Zn^2+^ ions with ICG in anhydrous methanol solution, then transferring it into a methanol solution containing methylimidazole, followed by stirring for 1 h and centrifugation (Wang K.-F. et al., 2023). To further improve the circulation time and tumor accumulation of ICG, red blood cell membranes (RBCM) were extracted to coat the material through electrostatic adsorption, resulting in ICG@RB-MOF (Figure 3A). It was observed that this multifunctional material, under 808 nm laser irradiation, could exert photothermal effects and inhibit the proliferation of pancreatic cancer cells (Figure 3A). CLSM experiments showed that surface modification with red blood cell membranes effectively promoted the uptake of the material (Figure 3B). The CCK-8 kit was used to assess cytotoxicity of ICG@RB-MOF, and results shown in Figure 3C indicate that coating with red blood cell membranes led to enhanced cytotoxicity because of higher uptake. Given the excellent fluorescence imaging properties of ICG, the authors conducted experiments on the distribution and metabolism of the material in mice. It was found that 24 h after intravenous injection, the red blood cell membrane-coated MOF material could more significantly accumulated in tumor tissues (Figure 3D). Subsequent *in vivo* treatment results also demonstrated that the ICG@RB-MOF + 808 nm laser irradiation group had the smallest tumor weight, indicating the best therapeutic effect (Figure 3E). This study demonstrates that by rationally utilizing MOFs to carry ICG molecules, effective treatment outcomes can be achieved, even for challenging diseases like pancreatic cancer. Moreover, the use of RBCM-encapsulated materials in this system not only enhances the stability of the materials but also improves their targeting ability toward cancer cells. However, recent studies have demonstrated that RBCM surface-modified with aptamers exhibit superior cancer cell targeting efficiency (Zou et al., 2024b). Alternatively, the direct use of cancer cell membranes for encapsulation is another option, but the safety of cancer cell membranes remains a critical concern that warrants further attention (Zou et al., 2024b).

## 4 Bimetallic MOFs-Loaded ICG for phototherapy

**FIGURE 4:**
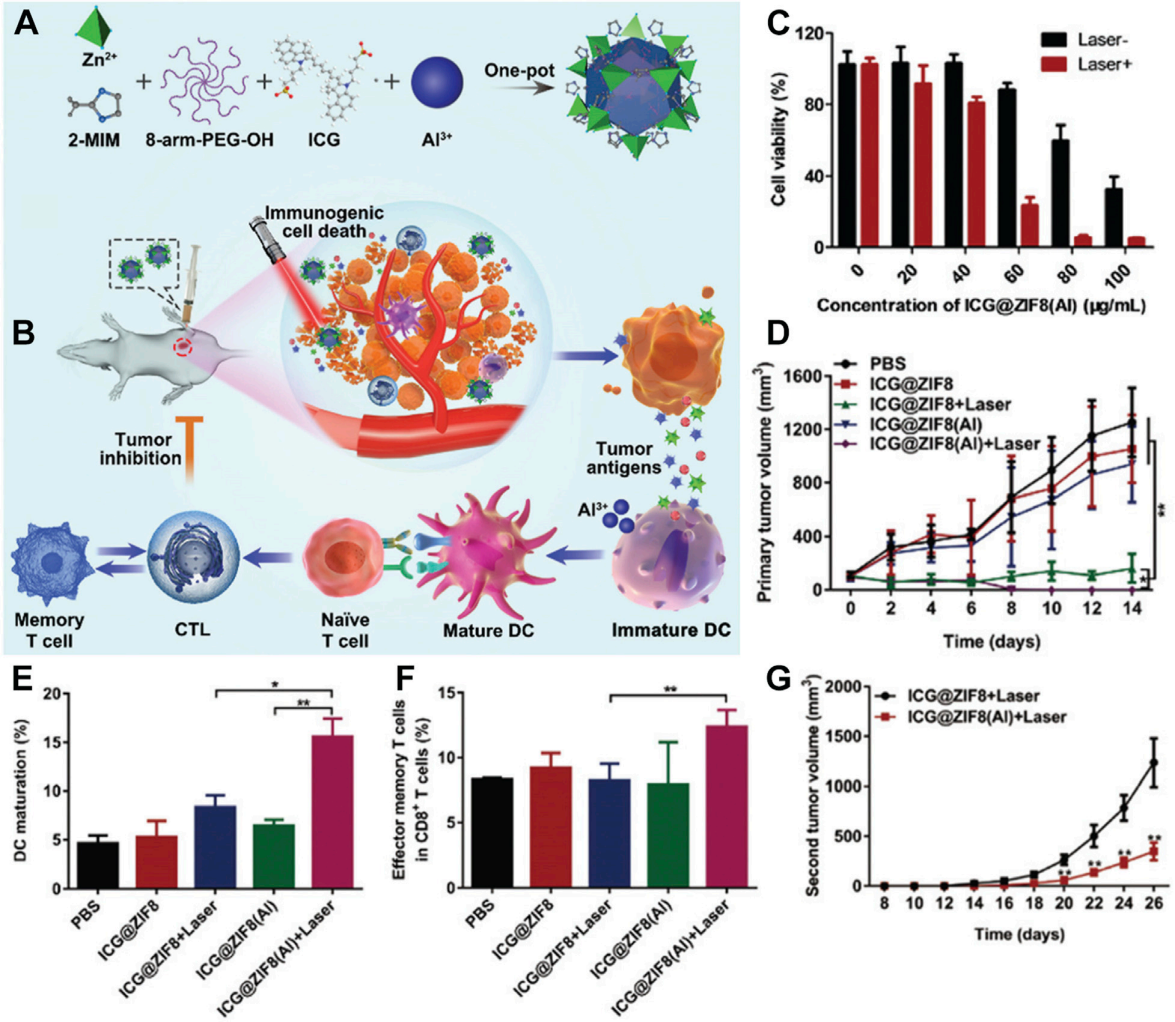
**(A)** Preparation of ICG@ZIF8(Al) NPs and **(B)** its photothermal-immunotherapy application. **(C)** Cytotoxicity of ICG@ZIF8(Al) NPs with or without laser irradiation. **(D)** Tumor volume changes after various treatments. **(E)** Matured DCs proportion after various treatments. **(F)** The change of effector memory T cells after various treatments. **(G)** Tumor volume changes of rechallenged tumors. Reproduced with permission from (Li A. et al., 2022). Copyright (2022), The Royal Society of Chemistry.

PTT not only kills malignant tumor cells by generating heat but can also induce immunogenic cell death (Duan et al., 2019; Li W. et al., 2019; Ma et al., 2019; Heshmati Aghda et al., 2020). Therefore, some researchers believe that ICG, while exerting photothermal effects under light exposure, may enhance the immune adjuvant effect, thereby improving the efficiency of malignant tumor suppression. Based on this idea, Yu et al. incorporated ICG and 8-arm-PEG-OH into a water solution containing methyl imidazole (Li A. et al., 2022). This mixture was then added to a mild solution containing zinc nitrate and aluminum chloride, stirred at room temperature, and subjected to centrifugation to obtain a multifunctional material doped with Al^3+^ ions, named ICG@ZIF8(Al) (Figure 4A) (Li A. et al., 2022). The authors demonstrated that this multifunctional material could effectively perform photothermal effects under 808 nm laser irradiation and enhance the immunoadjuvant effect of aluminum through the mechanism of immunogenic cell death induced by photothermal treatment, achieving a synergistic photothermal-immunotherapy effect (Figure 4B). In specific experiments, the inhibitory effect of the material on 4T1 cancer cells was verified using the MTT assay (Chen et al., 2020; Zhao et al., 2021; Yapeng et al., 2022). As shown in Figure 4C, at a concentration of only 60 μg/mL and under light exposure, the survival rate of 4T1 cells was below 50%. *In vivo* 4T1 tumor suppression experiments also showed that the group treated with ICG@ZIF8(Al) + laser had the smallest tumor volume (Figure 4D), attributed to the successful combination of PTT and immunotherapy. To further investigate the mechanism by which the material activates immunotherapy, flow cytometry was used to assess its immunological effects. As shown in Figure 4E, the ICG@ZIF8(Al) + laser group significantly promoted the maturation of dendritic cells (DCs), resulting from the combined action of PTT and the immune adjuvant. Compared to the ICG@ZIF8 group, the ICG@ZIF8(Al) group also produced more effector memory T cells (Figure 4F). Therefore, the authors conducted a re-challenge tumor suppression experiment by implanting tumors on one side and treating them, followed by observing the proliferation of cancer cells on the other side of the same mouse. The results showed that the tumor volume in the ICG@ZIF8(Al) + laser group was significantly smaller than that in the ICG@ZIF8 + laser group (Figure 4G), indicating that the materials prepared in this system can effectively activate the immune function of mice and suppress the proliferation of re-challenged tumors. This system is simple and effective, providing new insights into the application of MOF-loaded ICG. Furthermore, in this system, the weight percentage of Al^3+^ ions in the nanomedicine formulation is only 0.4%, which is relatively low. This may be attributed to the presence of Al^3+^ in the form of AlO(OH), potentially compromising the efficacy of aluminum as an immunological adjuvant. This suggests that the use of ZIF-MOF as a carrier for Al^3+^ may not be the optimal choice. Existing literature has demonstrated that aluminum can serve as a vaccine adjuvant in various forms, including aluminum hydroxide, aluminum phosphate, and amorphous aluminum hydroxyphosphate sulfate (Moni et al., 2023). The loading efficiency of different carriers for these forms may vary significantly. Therefore, the nano-delivery of aluminum-based immunological adjuvants remains a topic worthy of further investigation.

## 5 ZIF-8 loaded ICG for combined PDT and CDT

**FIGURE 5:**
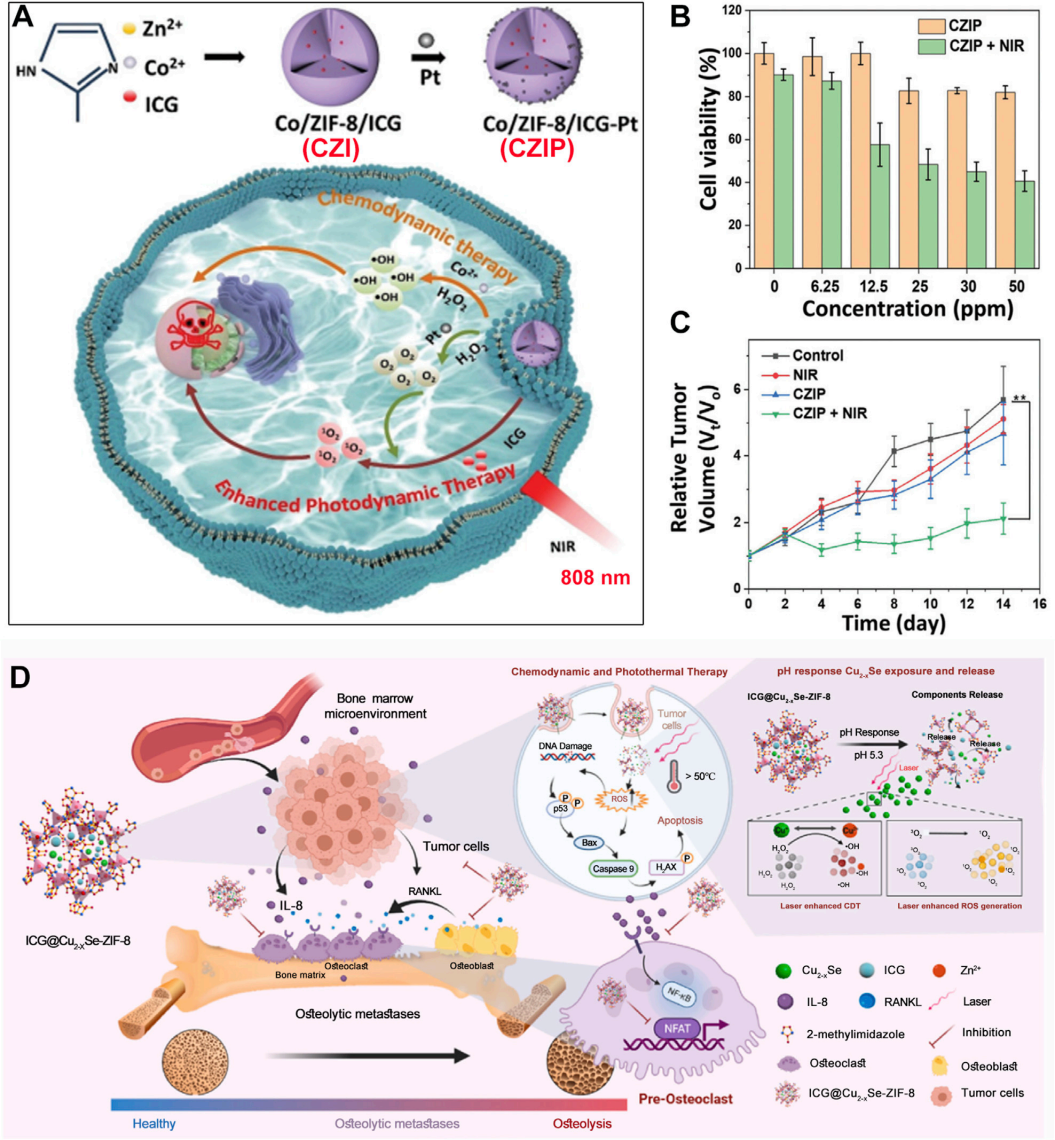
**(A)** Preparation process of CZIP and its antitumor mechanism. **(B)** Cytotoxicity after incubation with CZIP. **(C)** Tumor volume changes after different treatments. Reproduced with permission from (Jiang F. et al., 2022). Copyright (2022), The Royal Society of Chemistry. **(D)** Chemodynamic therapy and phototherapy application of ICG@Cu_2-x_Se-ZIF-8. Reproduced with permission from (Zou et al., 2022). Copyright (2022), Elsevier.

In addition to photothermal effects, ICG molecules can also efficiently generate ^1^O_2_ under light exposure, increasing oxidative stress within cancer cells, ultimately leading to apoptosis or necrosis. However, the hypoxic microenvironment of cancer cells has always been a significant factor limiting the efficiency of PDT (Li et al., 2018; Wei et al., 2018; Shen et al., 2021; Wan et al., 2021; Zheng et al., 2022). To mitigate the hypoxic conditions in cancer cells, researchers have found that platinum nanoparticles (Pt NPs), which are stable catalysts, can catalyze H_2_O_2_ to produce O_2_ within cancer cells, thereby enhancing PDT efficacy (Hao et al., 2020; Xu et al., 2020; Yang et al., 2020; Garcia-Peiro et al., 2022; Wang X. et al., 2022). Moreover, bimetallic-functionalized ZIFs can be prepared to enable MOF carriers to catalyze H_2_O_2_ into highly oxidative hydroxyl radicals through a CDT mechanism. Based on this concept, Lin et al. incorporated zinc nitrate and cobalt nitrate into a methanol solution containing methyl imidazole and ICG, stirred at room temperature, and centrifuged to obtain Co/ZIF-8/ICG composite (named CZI) (Jiang F. et al., 2022). Subsequently, by stirring a solution of CZI with Pt NPs coated with polyvinylpyrrolidone (PVP) at room temperature, they obtained Co/ZIF-8/ICG-Pt composite (named CZIP) (Figure 5A). Mechanistic validation revealed that after cellular uptake, CZIP could release Pt NPs and ICG molecules. Pt NPs catalyzed H_2_O_2_ to produce O_2_, enhancing the photodynamic effect of ICG under 808 nm laser irradiation. Additionally, Co^2+^ ions could catalyze H_2_O_2_ to produce hydroxyl radicals via a Fenton-like reaction, killing cancer cells (Figure 5A). The authors used the CCK-8 assay to evaluate the phototherapy efficacy of CZIP (Cheng Q. et al., 2021; Hu Y. et al., 2021). Results showed that under 808 nm laser irradiation, only 25 μg/mL of CZIP was required to cause more than 50% death of HeLa cells (Figure 5B). *In vivo* experiments suppressing U14 cancer cells in mice also demonstrated that CZIP, under 808 nm laser irradiation, could inhibit tumor growth through the combined mechanisms of PDT and CDT (Figure 5C). This system provides an effective design for enhancing the PDT application of ICG. Besides incorporating Co^2+^ ions into the ZIF structure, another approach involves using redox-active metal-based MOFs as carriers for ICG. For example, Hu et al. developed Ce^3+^ and TCPP photosensitizer-based MOFs. Ce-TCPP MOFs contain Ce^3+^, which has CDT capabilities, while TCPP generates PDT effects under 660 nm light (Lei et al., 2025). Utilizing this framework, ICG was loaded and Mn^2+^ ions was introduced to enhance the CDT process, achieving combined PDT, PTT, and CDT effects against B16 cancer cells. This system provides important insights for the combined antitumor applications of CDT and PTT. In these systems, the incorporation of cobalt and cerium metal endows the nanomedicine with chemical CDT functionality. However, it is important to note that the biocompatibility of cobalt and cerium may be inferior to that of essential trace elements in the human body, such as iron and manganese. Therefore, the dosage of cobalt and cerium should be carefully controlled. Additionally, both iron and manganese can also be doped into the ZIF-MOF to achieve CDT functionality (Dash et al., 2025).

Although Lin et al. focused solely on the photodynamic tumor suppression mechanism of ICG, during actual cell and animal experiments, they used a laser power intensity of 1.5 W/cm^2^ for 10 min, indicating that the photothermal effect of ICG contributed to its therapeutic outcomes (Jiang F. et al., 2022). Furthermore, besides the CDT effects of Co^2+^ ions, Cu^+^ ions are widely studied for their role in catalyzing the CDT process. PTT can effectively promote the efficacy of CDT, producing synergistic effects. Based on this background, Chen et al. first prepared monodisperse Cu_2-x_Se NPs by controlling nucleation rates (Zou et al., 2022). Then, Cu_2-x_Se NPs were added to a methanol solution containing methyl imidazole and ICG, followed by the addition of a methanol solution of zinc nitrate. After stirring at room temperature for 24 h, they obtained a multifunctional material comprising ICG and Cu_2-x_Se within a ZIF structure (ICG@Cu_2-x_Se-ZIF-8) (Figure 5D). This multifunctional material can degrade in the acidic environment of cancer cells, releasing Cu_2-x_Se and ICG. Cu_2-x_Se can further decompose into Cu^+^ and Cu^2+^ ions, where Cu^+^ ions catalyze the reaction of intracellular H_2_O_2_ to produce hydroxyl radicals, achieving a CDT effect. Additionally, under 808 nm laser irradiation, both Cu_2-x_Se and ICG exhibited photothermal effects. The photothermal effect enhanced the production of ^1^O_2_ and hydroxyl radicals, demonstrating the enhancement of CDT by photothermal effects (Figure 5D). Moreover, the Se element in Cu_2-x_Se materials was found to inhibit cancer cell proliferation by regulating Se proteins within cancer cells. Experimental results ultimately indicated that the multifunctional ICG@Cu_2-x_Se-ZIF-8 material could combat tumor cells in bone tissues through the combined effects of PTT and CDT (Figure 5D) and reduce the formation of osteoclasts to inhibit bone metastasis in breast cancer patients.

## 6 NIR-IIb-responsive MOF@ICG composite for combined PDT and PTT

**FIGURE 6:**
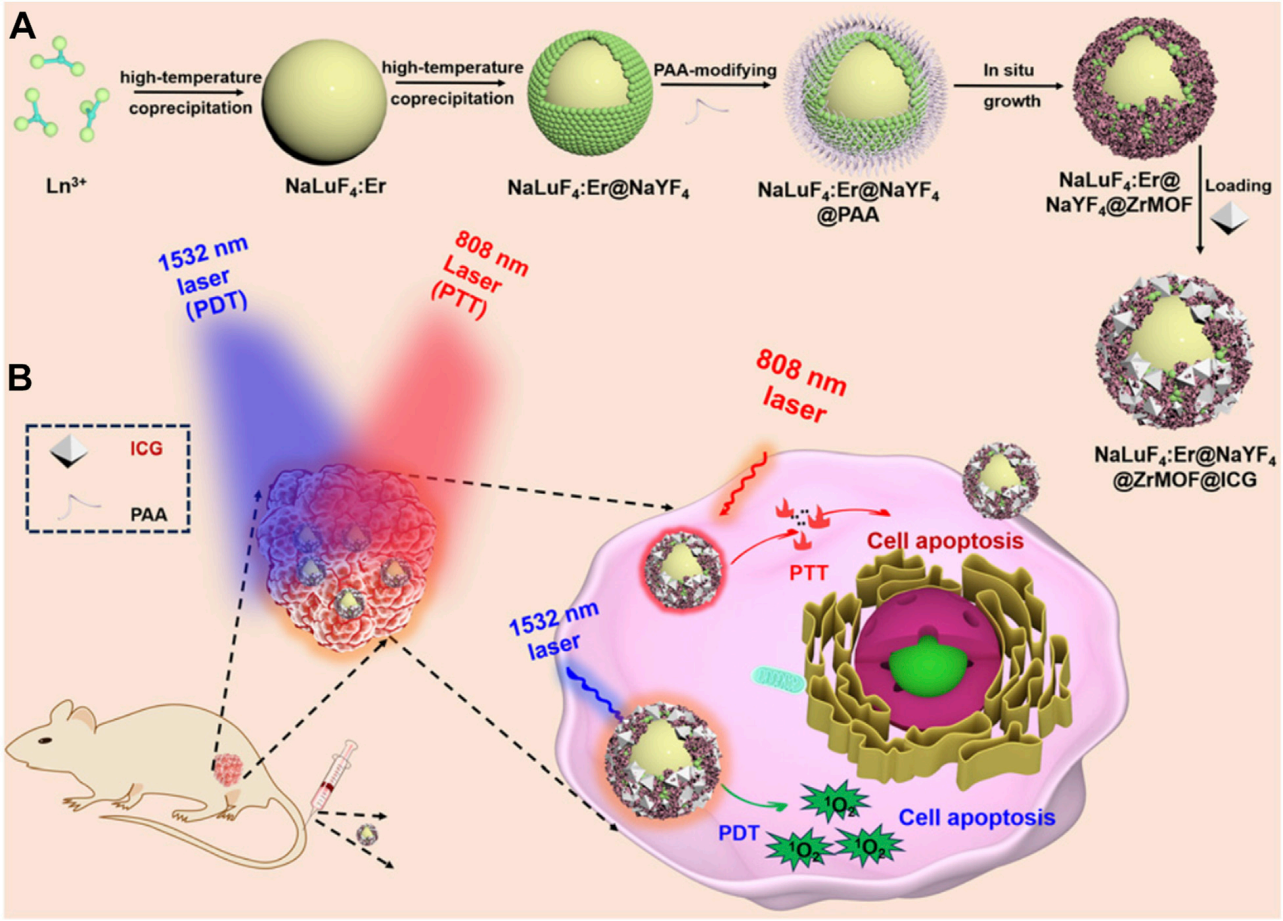
**(A)** Preparation process of UCNPs@ZrMOF@ICG nanocomposites and **(B)** their antitumor mechanism. Reproduced with permission from (Jiang et al., 2024). Copyright (2024), The Royal Society of Chemistry.

Although ICG can effectively generate photothermal effects and kill cancer cells under 808 nm laser irradiation, it is well known that ICG has poor photostability. This is because ICG itself can produce singlet oxygen under 808 nm light exposure, which oxidizes and damages its structure (You et al., 2017; Clutter et al., 2022). Moreover, despite 808 nm being in the near-infrared region, the penetration depth remains limited. Therefore, using excitation light sources with longer wavelengths and weaker tissue absorption might yield unexpected results and offer hope for treating disease in deeper tissues. For instance, light sources in the second near-infrared window (NIR-II, 1,400–1,600 nm) could provide more satisfactory penetration depths if used for photoactive materials (Jung et al., 2019; Chan et al., 2022; Wang Q. et al., 2023; Qin et al., 2024). Researchers have found that upconversion nanoparticles (UCNPs) prepared by doping with lanthanide elements may achieve absorption in the NIR-II region, and their upconverted emission spectra lie between 500 and 700 nm (Jiang et al., 2024). This wavelength range overlaps with the absorption spectrum of porphyrin photosensitizers. Based on this background, Li et al. prepared NaLuF4:Er@NaYF4 composite material using a two-step high-temperature co-precipitation method (Jiang et al., 2024). They then modified the surface with polyacrylic acid to make it hydrophilic. Subsequently, Zr-MOF constructed from Zr^4+^ and TCPP molecules was grown *in situ* on the surface of this material. Utilizing the pore structure of Zr-MOF, they loaded the ICG molecule, ultimately obtaining the NaLuF4:Er@NaYF4@ZrMOF@ICG composite (Figure 6A). The authors found that the UCNP component in this composite can absorb excitation light at 1,532 nm and emit primary peaks at 550 nm and 670 nm, suitable for exciting porphyrin photosensitizers to produce ^1^O_2_ for PDT. Under 808 nm laser irradiation, the ICG within the composite converts light energy into heat. Ultimately, this composite material achieved combined PDT and PTT treatment of HCT116 cancer cells under simultaneous excitation by 1,532 nm and 808 nm lasers (Figure 6B). This system provides new ideas for treating deeper tissue cancer cells and holds certain research significance. However, using two different excitation light sources might complicate the treatment process and increase costs. Notably, the use of a 1,532 nm excitation light source in this system is highly uncommon, suggesting that the translation of related research into clinical applications may face significant challenges.

## 7 MOFs-loaded ICG and chemotherapeutic agents for combined phototherapy and chemotherapy

**FIGURE 7:**
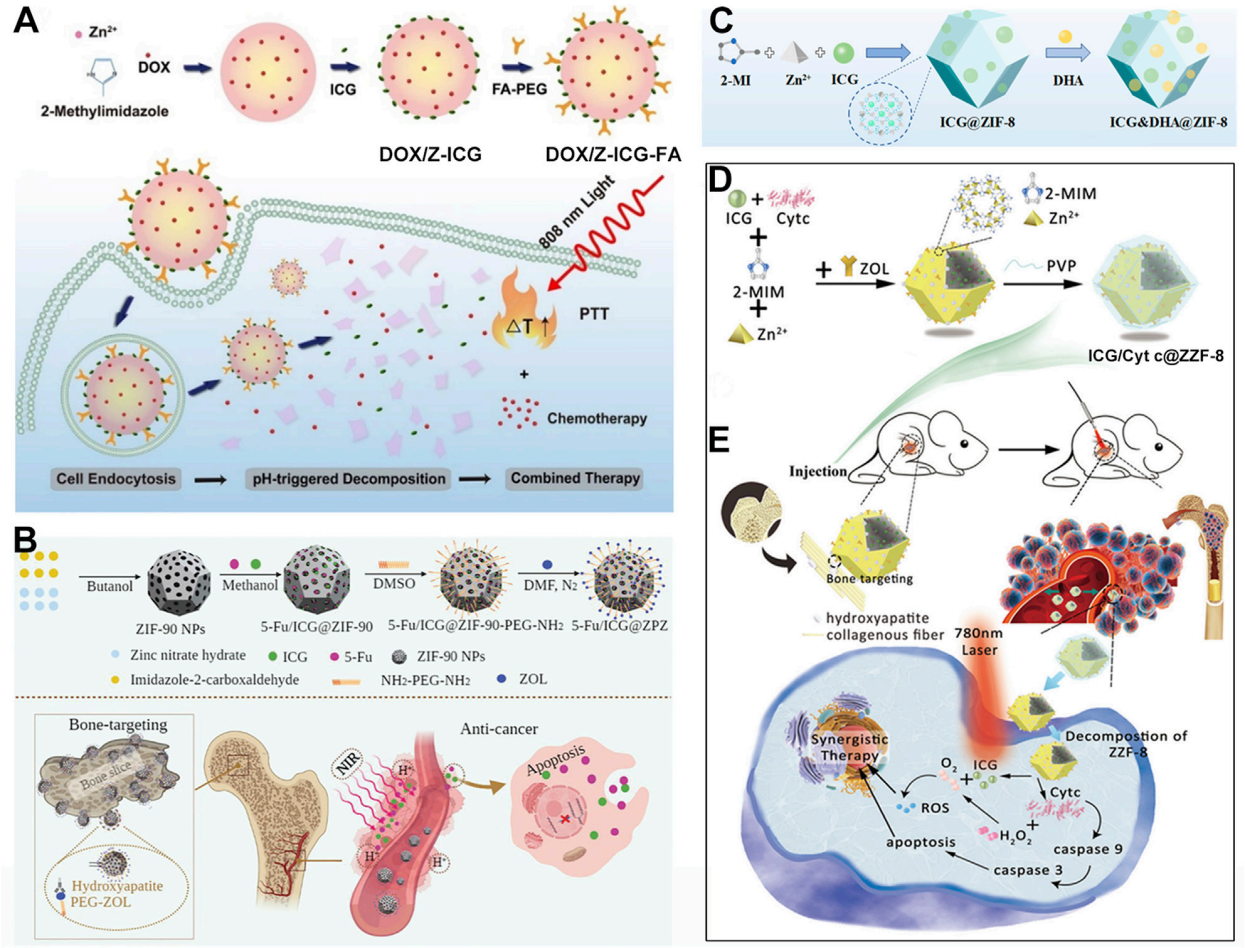
**(A)** Synthesis process of DOX/Z-ICG-FA and their antitumor application. Reproduced with permission from (Liu B. et al., 2022). Copyright (2022), Springer Vienna. **(B)** Preparation schematic and anticancer mechanism of 5-Fu/ICG@ZIF-90-PEG-ZOL. Reproduced with permission from (Ge T. et al., 2022). Copyright (2022), The Royal Society of Chemistry. **(C)** Preparation procedure of ICG&DHA@ZIF-8. Reproduced with permission from (Chen et al., 2022). Copyright (2022), MDPI (Basel, Switzerland). **(D)** Preparation schematic and **(E)** anticancer mechanism of ICG/Cyt c@ZZF-8@PVP NPs. Reproduced with permission from (Jiang Z. et al., 2022). Copyright (2022), The Royal Society of Chemistry.

In recent years, there has been increasing interest in using MOFs as carrier materials. The unique structure of MOFs not only allows them to load metal ions through the unsaturated coordination of ligands but also enables them to carry drug molecules via their pore structures or surface modifications. This versatility allows MOFs to efficiently load various functional drug molecules, such as chemotherapy drugs and photosensitizers, achieving combined therapeutic effects. The combination of photothermal therapy and chemotherapy has also been proven to be a highly effective cancer treatment modality (Tian R. et al., 2020). For instance, Chu et al. used a one-pot synthesis method to prepare nanomaterials encapsulating DOX and then modified the surface with ICG and folic acid-linked PEG molecules to obtain the multifunctional material DOX/Z-ICG-FA (Figure 7A) (Liu B. et al., 2022). With the assistance of the active targeting of folic acid (Kuang et al., 2023a), DOX/Z-ICG-FA can actively target cancer cells and be effectively internalized by cancer cells. After cellular uptake, the acidic environment of cancer cells causes the ZIF structure to decompose, releasing DOX and ICG. Under 808 nm laser irradiation, the photothermal effect of ICG combines with the chemotherapeutic action of DOX, effectively inhibiting the proliferation of MCF-7 cancer cells. This system leverages the modifiability of MOFs to design simple yet effective NPs for combined PTT/chemotherapy, providing new avenues for treating malignant tumors. The system employs folic acid small molecule-modified materials to achieve active targeting. However, recent clinical studies have shown that clinical targeting strategies based on folic acid have consistently failed, primarily due to significant differences in the targeting efficacy of folic acid between *in vitro* and *in vivo* applications (Wang H. et al., 2020; Wang H. et al., 2022). Therefore, more scientific research data are needed to advance the clinical application of nanomedicines leveraging folic acid-targeting mechanisms for enhanced therapeutic effects.

Apart from carrying common chemotherapy drugs like DOX, MOFs can also load other small molecule chemotherapy drugs. 5-Fluorouracil (5-FU) is a chemotherapy drug that affects nucleic acid synthesis and metabolism in cancer cells. However, it faces issues of drug resistance and poor targeting. Therefore, a carrier is needed to improve its accumulation at the lesion site. Using MOFs as carrier materials to load 5-FU and photothermal agents can enhance drug delivery and inhibit tumor cell growth, especially for cancers prone to metastasis, such as bone cancer. For example, Yang et al. first obtained ZIF-90 NPs through ultrasonic treatment, vigorous stirring, and centrifugation (Ge T. et al., 2022). Then, they dispersed ICG, 5-FU, and ZIF-90 NPs in methanol solution, stirred and centrifuged to obtain the 5-FU/ICG/ZIF-90 composite (Figure 7B). To enhance bone-targeting capabilities, activated zoledronate (ZOL), which has a special affinity for bone, was modified onto the surface of 5-FU/ICG/ZIF-90 to produce the multifunctional 5-FU/ICG/ZPZ material (Figure 7B). With the assistance of zoledronate’s targeting ability, 5-FU/ICG/ZPZ can effectively target pathological areas within bone tissue. The 5-FU and ICG released from ZIF-90, can effectively inhibit the growth of MCF-7 cancer cells under 808 nm laser irradiation (Figure 7B). Through rational design, this system combines the chemotherapeutic effect of 5-FU with the PTT effect of ICG, offering new hope for treating highly metastatic and drug-resistant bone cancers.

Besides loading DOX and 5-FU, ZIF-MOFs can also carry dihydroartemisinin (DHA) as a chemotherapy drug and ICG as a photothermal agent for the combined treatment of liver cancer cells. Li et al. encapsulated ICG into ZIF-MOF using a one-pot method and then absorbed DHA molecules under mild conditions to prepare the multifunctional ICG&DHA@ZIF-8 nanoplatform (Figure 7C) (Chen et al., 2022). In an acidic cellular environment, ICG and DHA are released. Subsequently, under 808 nm laser irradiation, the PTT and chemotherapy effects are triggered. These combined effects effectively inhibit the growth of HepG2 cancer cells. This system provides new insights into the biomedical applications of MOF/ICG based nanomedicine.

Moreover, besides small molecule chemotherapy drugs, MOFs can also load large protein molecules. Some types of MOFs, such as the ZIF series, have mild synthesis conditions, allowing direct loading of proteins without causing deactivation due to harsh conditions or additional bonding reactions. For example, Jiang et al. used a one-pot mineralization method in a PVP aqueous solution containing methyl imidazole, ICG, and cytochrome c (Cyt c) (Jiang Z. et al., 2022). Slowly adding zinc nitrate solution under vigorous stirring followed by adding an aqueous solution of zoledronate resulted in the ICG/Cyt c@ZZF-8 composite material (Figure 7D). Results showed that zoledronate endows the material with the ability to target bone cancer cells. After targeting, under 780 nm light irradiation, ICG/Cyt c@ZZF-8 produces ROS to inhibit the growth of 4T1 cancer cells. Additionally, the released Cyt c can induce caspase activation, leading to programmed cancer cell death, and act as a catalase to decompose intracellular H_2_O_2_ into O_2_. The generated O_2_ not only relieves the hypoxic solid tumor environment but also enhances the PDT efficiency of ICG (Figure 7E). By leveraging the mild synthesis conditions of ZIF-MOFs, this system achieves the combined application of ICG phototherapy and co-loaded anticancer protein drugs, providing new ideas for treating troublesome diseases.

## 8 MIL-100 MOFs-Loaded ICG for combined chemo-photothermal therapy

**FIGURE 8:**
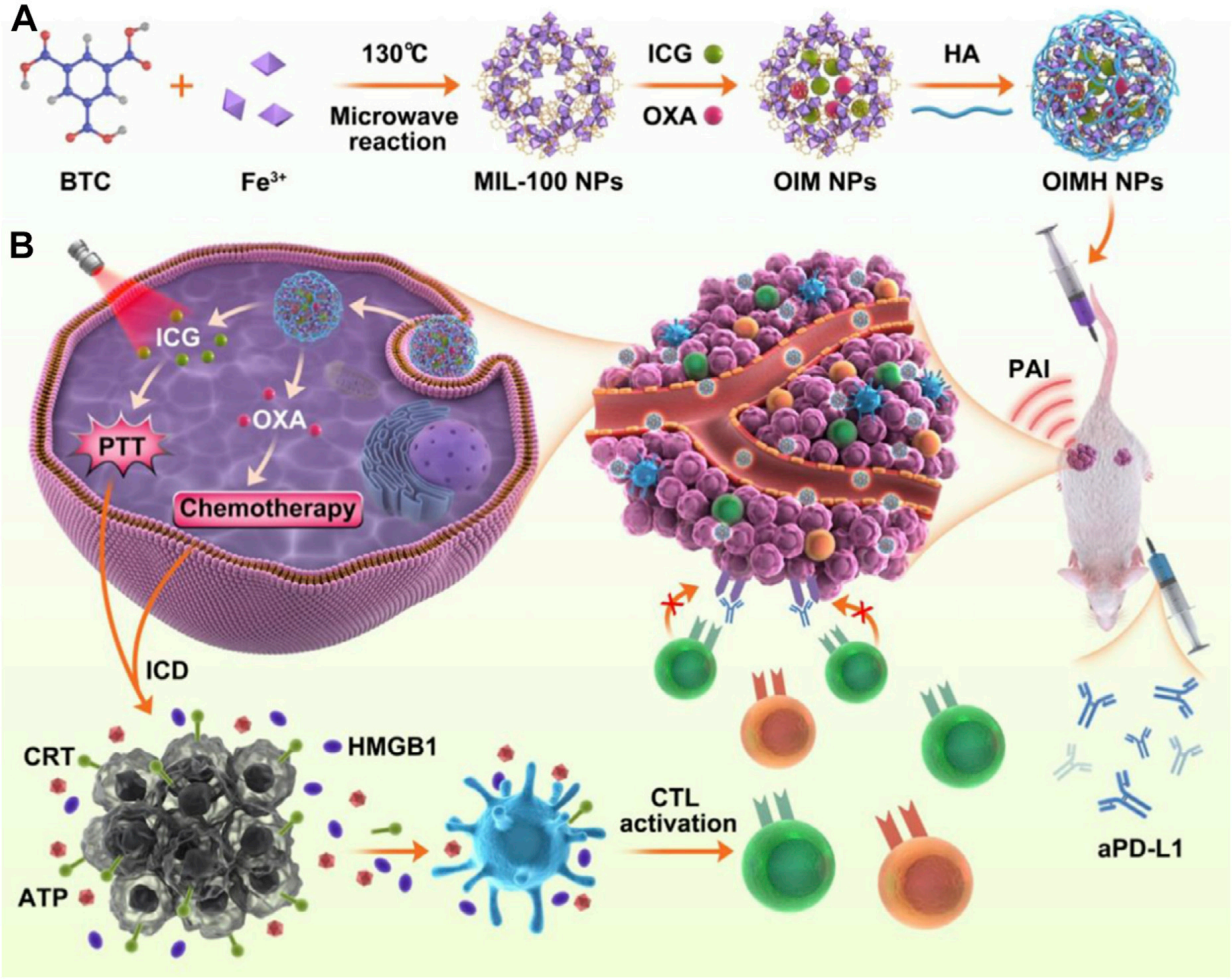
**(A)** Preparation procedures of OIMH NPs and **(B)** PAI-guided anticancer mechanism. Reproduced with permission from (Liu H. et al., 2022). Copyright (2022), Elsevier.

Although MOFs can serve as carriers for chemotherapy drugs and photoactive materials like ICG, achieving combined chemo-photothermal therapy, their long circulation stability and the resulting accumulation of drugs in tumor cells through the EPR effect may still be insufficient. The large specific surface area of MOFs allows for various polymers, such as hyaluronic acid (HA) to be surface-modified, thereby improving the stability of MOF materials and enhancing their accumulation in cancer cells. The combination of HA with photothermal agents has been demonstrated to enhance their biocompatibility and therapeutic efficacy (Guo et al., 2023). For example, Liu et al. prepared MIL-100 NPs using a microwave heating method via the coordination reaction between benzene-1,3,5-tricarboxylic acid (BTC) and Fe^3+^ ions (Figure 8A) (Liu H. et al., 2022). Subsequently, MIL-100 NPs were mixed with oxaliplatin (OXA) and ICG by stirring and then centrifuged to obtain OXA and ICG-loaded OXA/ICG/MIL-100 nanoplatform (referred to as OIM NPs). To further enhance the long circulation capability, HA was modified onto the surface of OIM NPs, yielding OIMH NPs (Figure 8A). Cell viability assays revealed that OIMH degrades in the weakly acidic microenvironment of cancer cells, releasing OXA and ICG. Under 808 nm laser irradiation, ICG molecules generate photothermal effects and photoacoustic imaging capabilities (Figure 8B). OXA exerts its chemotherapeutic effect. Moreover, both PTT and chemotherapy can induce immunogenic cell death (ICD), activating the body’s immune response. However, this activated immune function alone is often insufficient to effectively inhibit cancer cell proliferation. In recent years, immunotherapy has attracted considerable attention from researchers worldwide, with various types being extensively studied. Among these, immune checkpoint blockade therapy is one of the widely researched approaches. Therefore, to enhance the combined efficacy of this system, the authors introduced anti-PD-L1 (aPD-L1) therapy. While single-agent immune checkpoint blockade therapy often fails to achieve satisfactory results in treating malignant colorectal cancer due to poor immune infiltration, the authors found that ICD induced by PTT and chemotherapy significantly enhances the effectiveness of immune checkpoint blockade therapy by sensitizing cancer cells. Through rational design, this system achieved combined chemo-photothermal therapy and aPD-L1 treatment against CT26 cancer cells (Figure 8B). This system provides new therapeutic strategies for colorectal cancer, a particularly challenging disease. Hyaluronic acid has been demonstrated to be a biocompatible polymer. However, it is important to note that the entry of large amounts of hyaluronic acid into the human body may potentially trigger an immune response due to structural alterations (Fillit et al., 1986).

## 9 Target peptide-guided MOF@ICG/DOX for combined photo-chemotherapy

**FIGURE 9:**
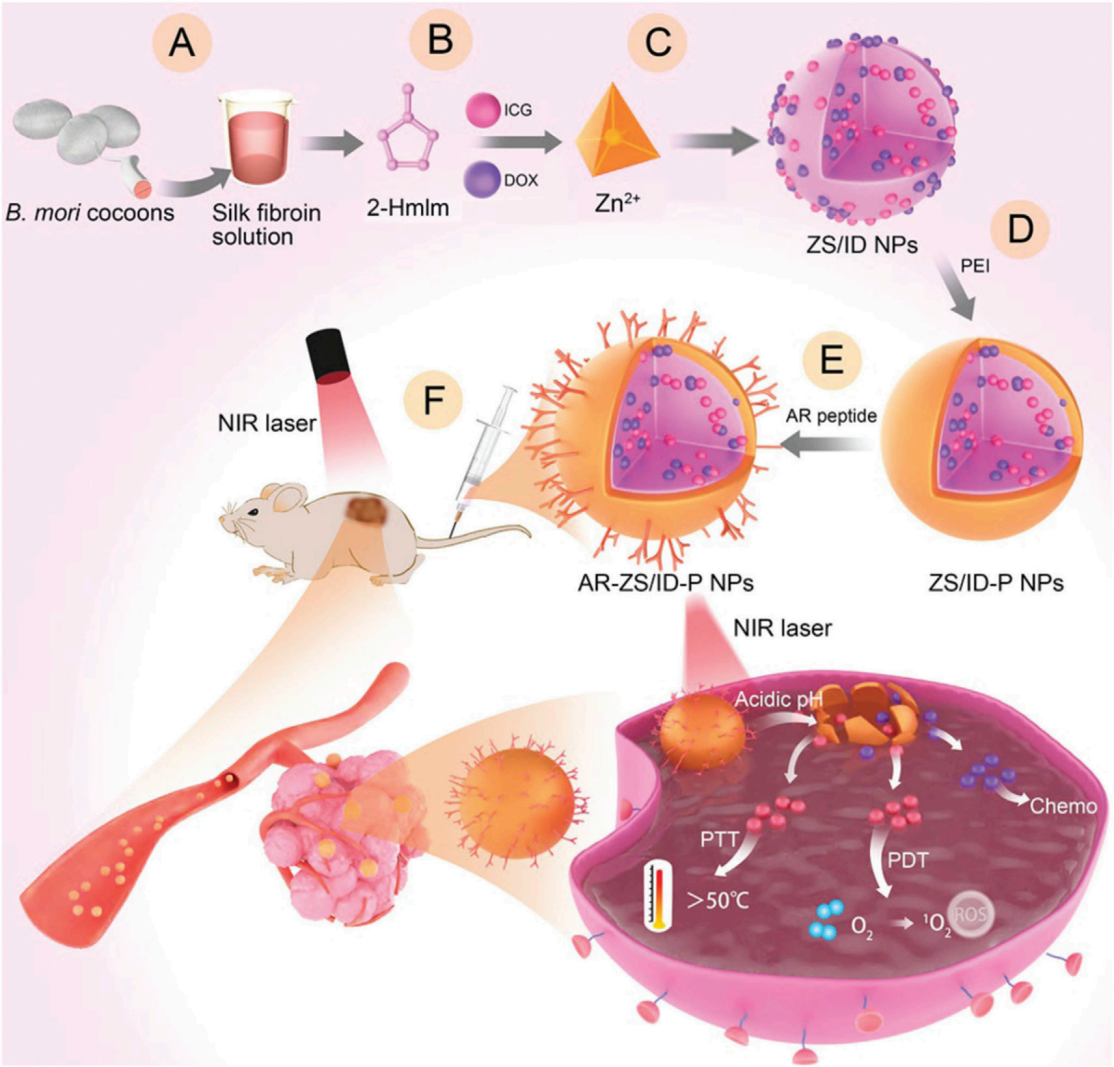
Preparation routes of AR-ZS/ID-P NPs and their anticancer mechanism. **(A)** Silk fibroin (SF) was used as a biotemplate, **(B)** addition of DOX and ICG, **(C)** preparation of ZS/ID NPs by one-pot process, **(D)** preparation of ZS/ID-P NPs, **(E)** preparation of AR-ZS/ID-P NPs. **(F)** Schematic illustration for the antitumor application of AR-ZS/ID-P NPs. Reproduced with permission from (Chen et al., 2023a). Copyright (2023), John Wiley & Sons, Ltd.

Authoritative research data indicate that relying solely on the EPR effect for the accumulation of NPs at the lesion site often results in insufficient drug accumulation. Therefore, it is necessary to modify MOF carrier materials with active targeting functionalities to enhance their targeting of cancer cells. Besides small molecules like folic acid and HA, or cell membranes, certain peptides can also endow targeting mechanisms to MOF materials. Utilizing phage display technology represents a novel approach that effectively avoids individual variability issues that other targeting molecules may encounter when targeting the same type of tumor. For example, Yang et al. first extracted fibroin molecules (Figure 9A), mixed them with methyl imidazole, ICG, and DOX (Figure 9B), and then rapidly formed a material loaded with ICG and DOX using Zn^2+^ ions via a one-pot method, named ZS/ID NPs (Figure 9C) (Chen et al., 2023a). Subsequently, a polyethyleneimine (PEI) coating was applied to the surface of ZIF-MOF to enhance its dispersion stability and provide sites for subsequent peptide targeting modifications, resulting in ZS/ID-P NPs (Figure 9D). The authors then reacted breast tumor-targeting peptides with PEI to prepare AR-ZS/ID-P NPs (Figure 9E). To validate the effectiveness of these materials in inhibiting MCF-7 cancer cells, the AR-ZS/ID-P NPs were injected via the tail vein. Due to the homing effect of the AR peptide, the prepared nanomaterials could effectively accumulate at the site of MCF-7 cancer cells (Figure 9F). After uptake by the cancer cells, the weakly acidic environment caused the ZIF-MOF structure to degrade, releasing DOX and ICG molecules. Under 808 nm laser irradiation, ICG can produce combined photodynamic and photothermal effects, while DOX exerts its chemotherapeutic action. Therefore, this system leverages the enhanced targeting induced by the AR peptide to achieve a combination of chemotherapy, PDT, and PTT for the inhibition of MCF-7 cancer cell growth. This system provides a promising multifunctional therapeutic strategy based on MOF-loaded ICG and DOX, offering new insights into efficient drug delivery. By integrating targeted delivery through the AR peptide, this approach not only enhances the accumulation of drugs within cancer cells but also maximizes the therapeutic impact by combining multiple treatment modalities. The system utilizes tumor-targeting peptides screened through phage display technology, which can significantly enhance the specific targeting efficacy of the materials and offers unique advantages. However, it is important to note that peptides are prone to degradation by proteases in the biological environment, potentially compromising their stability (Fosgerau and Hoffmann, 2015). Additionally, non-specific binding may also reduce the targeting efficiency (Ruoslahti, 2012). Furthermore, whether the technology employed in this system can be scaled up for mass production remains a significant challenge, which poses substantial obstacles for clinical translation.

## 10 Multifunctional MOFs@ICG/DOX for combined phototherapy and chemotherapy

Although ZIF-MOFs can effectively load ICG and DOX to achieve combined phototherapy and chemotherapy, the ZIF structure typically serves merely as a carrier without contributing additional biological functions. Furthermore, some researchers believe that encapsulating different drug molecules in separate compartments during combination therapy may offer unique advantages. Considering the variety of MOFs, MIL and ZIF represent different types of MOFs. The common MIL series containing Fe elements can react with excess H_2_O_2_ in cancer cells to produce O_2_, thereby relieving the hypoxic microenvironment within solid tumors (Wu B. et al., 2020). Based on this, Wu et al. developed a core-shell dual-layer MOF as a multifunctional combined drug delivery system (Wu B. et al., 2020). Wu et al. first coordinated amino-substituted terephthalic acid with Fe^3+^ ions to form MIL-88 MOF material, then utilized its pore structure to load ICG molecules, obtaining MIL-88-ICG (Wu B. et al., 2020). Subsequently, by stirring a mixture of methyl imidazole and Zn^2+^ ions on the surface of MIL-88-ICG, they obtained a core-shell structured material, MIL-88-ICG-ZIF-8. Utilizing the pore structure of ZIF, they further loaded DOX chemotherapy drugs to obtain the final multifunctional material, MIL-88-ICG@ZIF-8-DOX. To enhance the effectiveness of transdermal administration, the authors mixed MIL-88-ICG@ZIF-8-DOX with hyaluronic acid (HA) to prepare microneedle patches. Subsequent therapeutic mechanism studies demonstrated that the developed system allows the degradation of the ZIF structure under the acidic microenvironment of cancer cells, releasing DOX. Moreover, laser irradiation accelerates the release of DOX, enhancing its chemotherapeutic effect. Meanwhile, ICG generates photothermal effects under 808 nm light irradiation. Fe^3+^ ions within the MIL series MOFs can react with excess H_2_O_2_ in cancer cells to produce O_2_, facilitating the generation of more ^1^O_2_ by ICG under laser irradiation for PDT. Thus, through the rational design of a dual-layer MOF carrier structure, the authors leveraged the ability of Fe^3+^ ions in MIL series MOFs to catalyze H_2_O_2_ into O_2_, thereby enhancing the PDT process. This ultimately achieved enhanced PDT/PTT/chemotherapy triple-combination treatment effects against 4T1 cancer cells.

**FIGURE 10:**
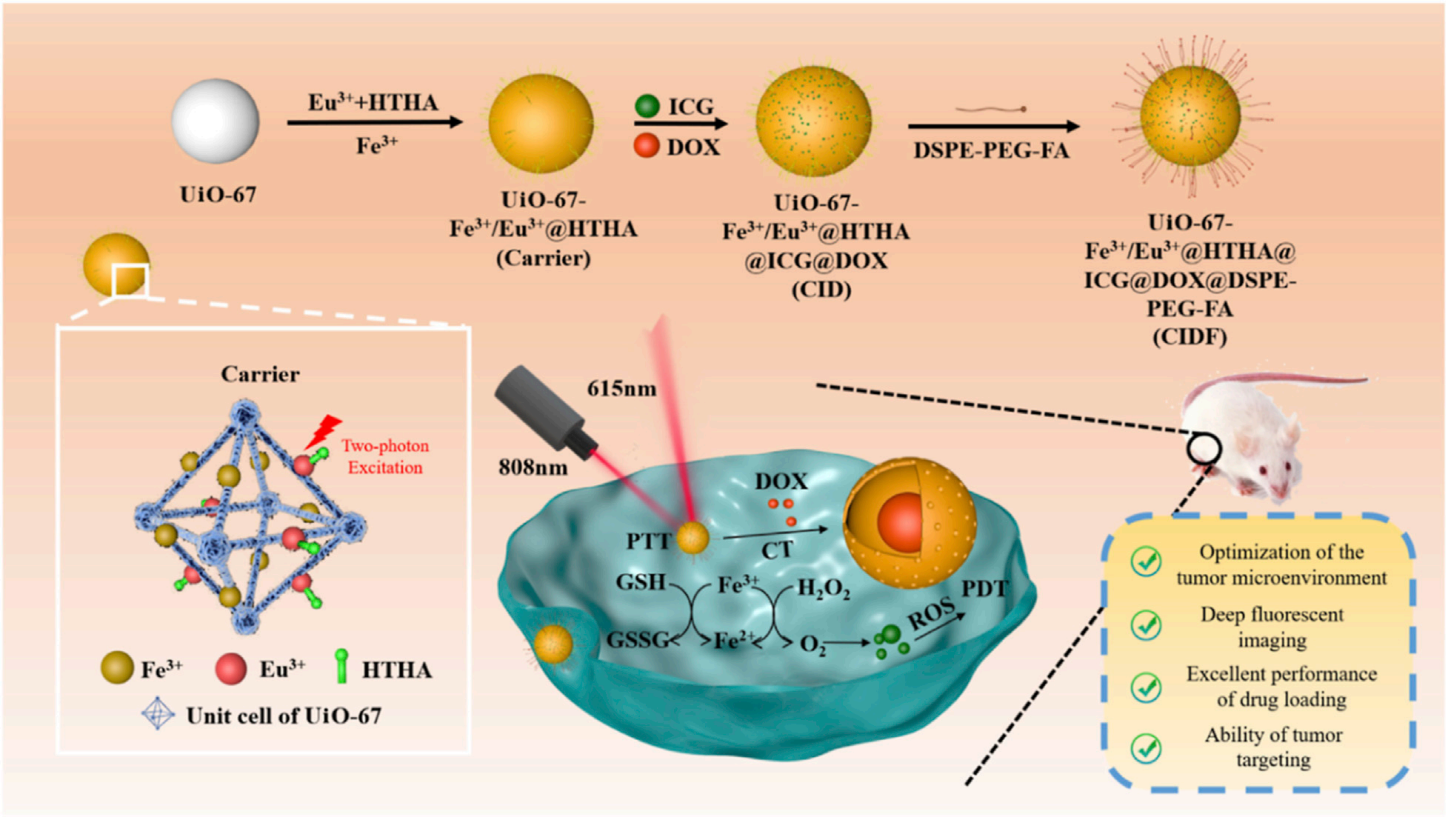
Preparation procedures of CIDF and its anticancer mechanism. Reproduced with permission from (Liu Y. et al., 2024). Copyright (2024), American Chemical Society.

In addition to enhancing phototherapy by generating O_2_ to relieve the hypoxic microenvironment, it is also possible to enhance phototherapy efficacy by regulating reductive molecules within cancer cells. For example, due to unique metabolic characteristics, cancer cells typically contain higher levels of the reductive small molecule-glutathione (GSH), which can neutralize ^1^O_2_ produced during PDT, thereby reducing its effectiveness. Therefore, developing a mechanism that not only generates O_2_ but also reduces intracellular GSH could more effectively enhance PDT efficacy. Based on this, Tang et al. used UiO-67 as a template and incorporated Fe^3+^ and Eu^3+^-HTHA into the structure of UiO-67 through coordination to obtain the carrier material UiO-67-/Fe^3+^/Eu^3+^@HTHA (Figure 10) (Liu Y. et al., 2024). This carrier was then used to load ICG and DOX drug molecules, resulting in UiO-67-Fe^3+^/Eu^3+^@HTHA@ICG@DOX (referred to as CID). To enhance the dispersion stability and targeting ability of CID towards cancer cells, the surface was modified with folic acid-conjugated DSPE-PEG, yielding the multifunctional composite material CIDF (Figure 10). Subsequent studies revealed that under 808 nm laser irradiation, HTHA molecules can transfer energy to excite Eu^3+^ ions, emitting fluorescence at 615 nm for imaging-guided treatment processes (Figure 10). Under 808 nm irradiation, ICG produces both photothermal and photodynamic effects. Fe^3+^ ions regulate the excessive H_2_O_2_ and GSH present in the cancer cell microenvironment through redox reactions between Fe^3+^/Fe^2+^, reducing GSH concentrations while catalyzing H_2_O_2_ to produce O_2_, thereby significantly enhancing PDT efficacy. This system attains the comprehensive development of multifunctional nanomedicine *via* rational design. It effectively harnesses the cancer cell microenvironment, exploits the two-photon conversion principles for deep-tissue fluorescence imaging, implements efficient drug-delivery strategies, and targets cancer cells precisely (Figure 10). This research provides a novel approach for the combination of exploiting the unique microenvironment within cancer cells and enhancing therapeutic efficacy.

## 11 ZIF-8 MOF-Loaded ICG for antibacterial applications

**FIGURE 11:**
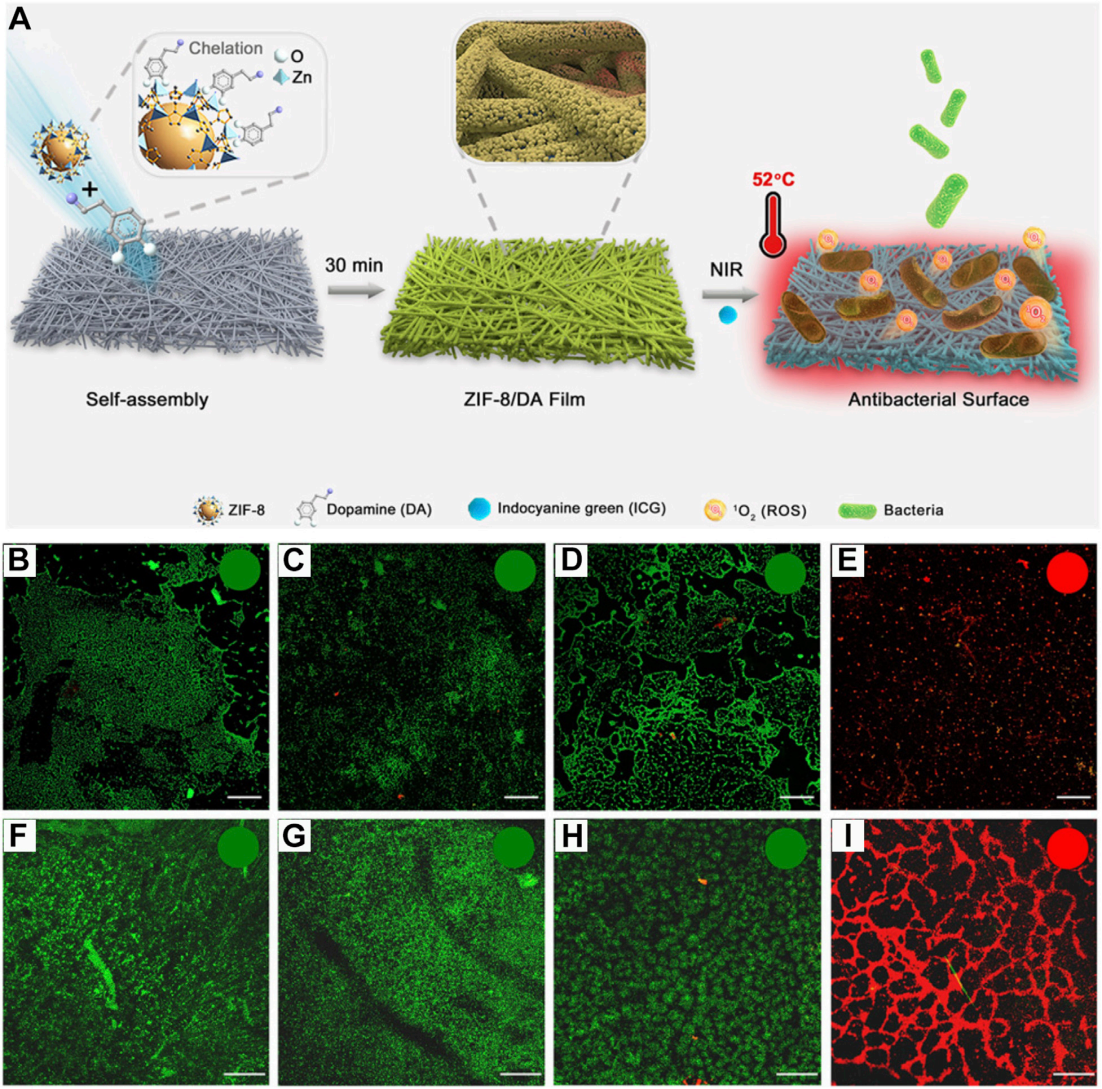
**(A)** Preparation procedures of ZIF-8/DA-0.5 and ZIF-8/DA-0.5/ICG samples. CLSM images of *E. coli* and *S. aureus* after various treatment including **(B/F)** FP-NIR, **(C/G)** ZIF-8/DA-0.5-NIR **(D/H)** ZIF-8/DA-0.5/ICG-Dark, and **(E/I)** ZIF-8/DA-0.5/ICG-NIR (scale bar: 100 μm). Reproduced with permission from (Gao et al., 2022). Copyright (2022), The Royal Society of Chemistry.

Bacterial infections have caused a great deal of disruption to human survival (Zhang et al., 2021; Lu et al., 2022). In recent years, research related to antibacterial properties has attracted the attention of many researchers (Li J. L. et al., 2019; Zhao et al., 2020; Wei et al., 2023). ZIF-MOF loaded with ICG can exhibit excellent PDT/PTT combined antitumor effects under 808 nm laser irradiation. Moreover, phototherapy also shows significant potential in the field of antibacterial applications (Yan et al., 2023). For instance, Ren et al. first prepared ZIF-8 NPs and then evaluated their binding capability with various substrate materials, including filter paper (FP), polypropylene nonwoven fabric (PP), cotton fabric, and medical gauze as model substrates (Gao et al., 2022). Results indicated that the binding of ZIF-8 to these substrates requires the assistance of dopamine (DA). The metal ions in ZIF-8 can chelate with the hydroxyl groups in the DA structure, facilitating the formation of a stable MOF layer on the substrate surface, named ZIF-8/DA Film (Figure 11A). The authors ultimately selected the assembly time of 0.5 h and a concentration ratio of ZIF-8 to DA of 1:1 for forming the ZIF/DA Film material. Given the porous structure of ZIF-8 MOF, the formed ZIF-8/DA film was subsequently used to adsorb the ICG molecule, resulting in the ZIF-8/DA-0.5/ICG platform (Figure 11A). Under 808 nm laser irradiation, this platform can convert light energy into heat with temperatures exceeding 50°C and produce ^1^O_2_ for PDT treatment. Based on these photophysical properties, the authors then tested the antibacterial efficacy of the composite. They further validated the inhibitory effect of the composite against *Escherichia coli* and *Staphylococcus aureus*. PET (polyethylene terephthalate) films were first formed in bacterial suspensions of both types of bacteria. The prepared film material was then placed over the PET surface and exposed to 808 nm laser for 10 min. Subsequently, live/dead bacterial staining was performed on the PET, followed by observation using CLSM. As shown in Figures 11B–E, compared to other control groups, the combination of ZIF-8/DA-0.5/ICG with laser irradiation resulted in the most significant bacterial death (red-stained regions), demonstrating the notable antibacterial performance of the film material. Similar effects were observed in the inhibition of *S. aureus* biofilms (Figures 11F–I). This system demonstrates that utilizing MOF materials to load ICG molecules can effectively perform antibacterial treatments, providing new routes for developing medical antibacterial materials. The system employs ICG as a phototherapy agent for antibacterial purposes, achieving some promising results. However, it is important to note that the photostability of ICG may limit the repeated use of the antibacterial agent and its ability to maintain efficacy over extended periods. On the other hand, while the system inhibits bacterial proliferation through phototherapy, repeated light exposure may lead to the selection of light-resistant bacterial strains (Hu et al., 2022; Surur et al., 2024). Additionally, batch-to-batch inconsistency in the oxidative polymerization of dopamine during large-scale production could also affect the therapeutic efficacy of the material. Therefore, practical applications of this system still face significant challenges.

## 12 PCN-224 MOF loaded ICG for phototherapy toward Alzheimer’s disease

**FIGURE 12:**
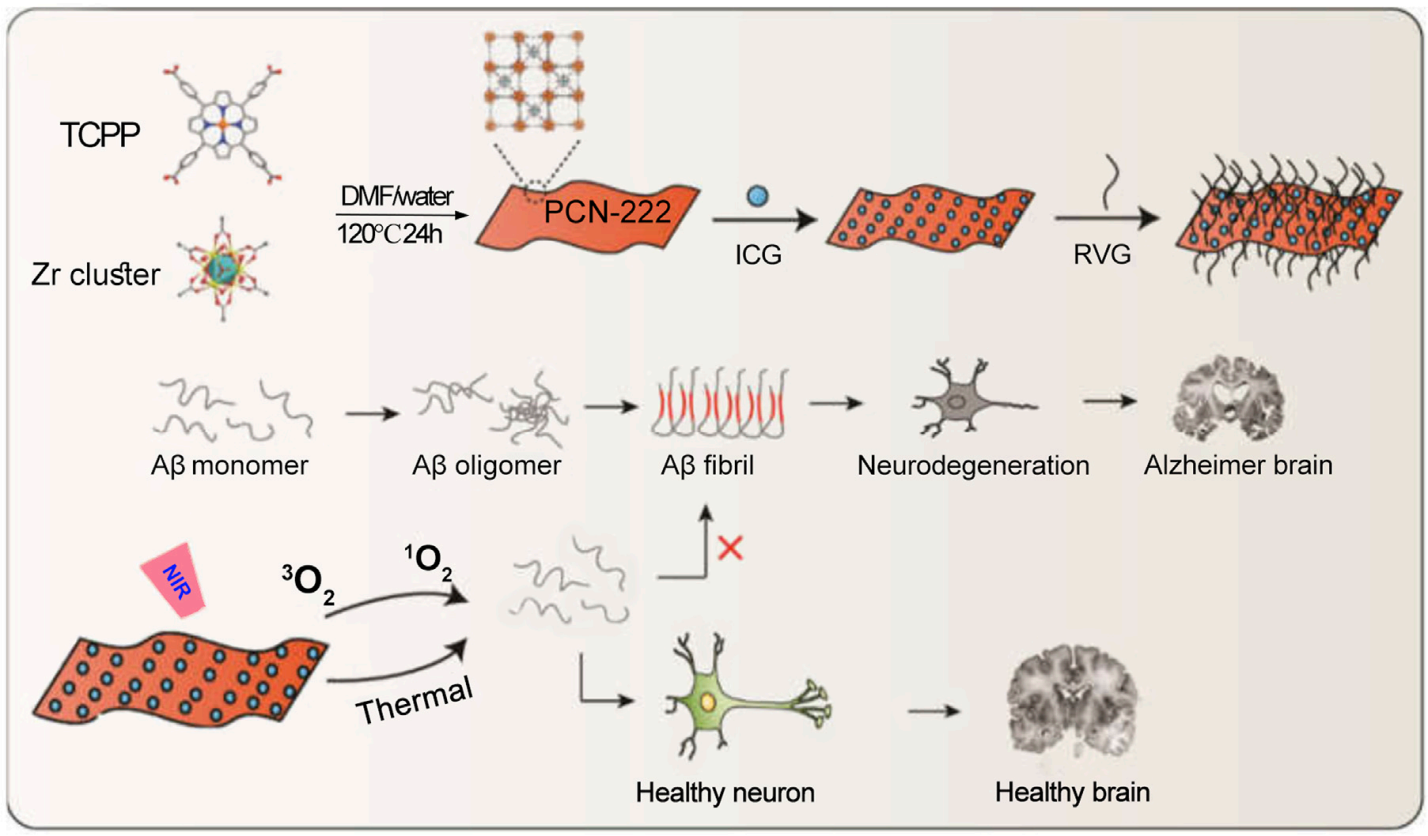
Synthesis schematic of PCN-222@ICG and the mechanism of phototherapy-induced inhibition of Aβ aggregation. Reproduced with permission from (Wang J. et al., 2022). Copyright (2022), MDPI (Basel, Switzerland).

Alzheimer’s disease is an age-related disorder that has significantly disrupted the quality of life of humans, especially the elderly (Mahaman et al., 2019). Various treatment approaches for Alzheimer’s disease have been extensively studied (Gong et al., 2021; Xu et al., 2022a; Kuang et al., 2023b). Research evidence indicates that the excessive production of extracellular amyloid-beta (Aβ) leads to the formation of oligomeric structures, which subsequently misfold into fibrillar Aβ plaques (Salahuddin et al., 2016; Chen et al., 2017; Mroczko et al., 2018; Hillen, 2019). These Aβ fibrils cause synaptic toxicity and neuronal degeneration, contributing to neurodegenerative diseases such as Alzheimer’s disease. Studies have shown that PDT can effectively disrupt the aggregated structures of Aβ. Therefore, Yang et al. developed a PCN-224 MOF composed of Zr^4+^ and TCPP (tetrakis(4-carboxyphenyl)porphyrin) (Wang J. et al., 2022). Utilizing the pore structure of PCN-224, they loaded the ICG molecule to obtain PCN-224@ICG. To enhance the targeting ability of the photoactive material towards brain lesions, they further modified the PCN-224 MOF with a brain-targeting peptide (RVG), resulting in the final composite: PCN-224@ICG@RVG (Figure 12). Subsequently, the authors verified that the brain-targeting peptide enhanced the material’s penetration through the blood-brain barrier (BBB). Under 808 nm laser irradiation, ICG performs PDT, generating ROS that oxidatively disrupt the aggregated Aβ. Meanwhile, the heat generated by the photothermal effect of ICG increases the instability of Aβ aggregates, thereby facilitating the structural disruption of Aβ plaques (Figure 12). This system provides new options and strategies for the preparation of phototherapy nanomedicines for Alzheimer’s disease and further expands the biomedical applications of MOFs/ICG nanomedicine. The system employs RVG peptides to enhance the targeting ability of nanomedicine and promote BBB penetration. However, it is important to note that the extent of BBB integrity damage varies among Alzheimer’s disease patients, which may influence the delivery efficiency of nanoprobes (Alkhalifa et al., 2023). On the other hand, the penetration depth of 808 nm laser light through skull-covered brain tissue is limited (Wu X. et al., 2020). Therefore, the development of noninvasive deep-tissue irradiation techniques is necessary to facilitate the clinical application of related drugs.

## 13 Conclusion

This review summarizes the research efforts involving MOFs as carrier materials for loading ICG molecules to achieve phototherapy in combination with other drug molecules for antitumor, antibacterial, and anti-Alzheimer’s disease treatments. The insights provided can guide the development of ICG-based nanomedicines by enhancing their circulation stability and targeting efficiency at disease sites. These studies demonstrate that ICG, as a multifunctional organic molecule, not only facilitates near-infrared fluorescence imaging, photothermal imaging, and photoacoustic imaging but also enables minimally invasive PDT and PTT. The integration of ICG with MOF carriers further underscores its significant potential for clinical applications.

However, several challenges remain with ICG. Firstly, ICG can generate singlet oxygen under light exposure, which may oxidize its own structure, making its photostability a critical limitation for broader application. Secondly, the PDT mediated by ICG relies on converting oxygen to singlet oxygen; thus, the hypoxic microenvironment of solid tumors limits the effectiveness of ICG’s photodynamic capabilities. Thirdly, the main absorption peak of ICG is around 780 nm, a wavelength that still struggles to penetrate deeper into tissue lesions. Furthermore, the pore size and internal structure of MOFs need to be carefully designed to accommodate the size and shape of ICG molecules. An ideal MOF structure should be able to encapsulate ICG efficiently while ensuring its stability and activity during storage and transport. Additionally, to enhance therapeutic efficacy and minimize side effects, the design of MOFs should incorporate specific stimuli-responsive release mechanisms, such as triggers based on pH, redox status, or enzyme sensitivity. Finally, Although the combination of MOFs with ICG shows great promise, the multifunctionality of MOF carriers endows the nanosystem with additional functionalities. However, the incorporation of these new functionalities may potentially increase the toxicity of the system. Therefore, more data from comprehensive toxicity assessments are needed to support the further preclinical application of the MOF-ICG system. Another key point is that although ICG has already been approved by the FDA, the safety of MOF carriers-such as their potential to trigger immune responses or cause long-term toxicity-remains a challenge for the clinical application of the MOF-ICG system. Other limiting factors include scalability of production, storage stability, and cost. Further research and more comprehensive data are essential to address these issues and facilitate the clinical translation of the MOF-ICG system. Addressing these issues will be crucial for advancing the biomedical applications of MOF-ICG systems.
